# Assessment of Toxic Pyrrolizidine and Tropane Alkaloids in Herbal Teas and Culinary Herbs Using LC-Q-ToF/MS

**DOI:** 10.3390/foods12193572

**Published:** 2023-09-26

**Authors:** Zinar Pinar Gumus

**Affiliations:** Central Research Test and Analysis Laboratory Application and Research Center (EGE-MATAL), Ege University, 35100 Izmir, Turkey; zinar.pinar.gumus@ege.edu.tr

**Keywords:** pyrrolizidine alkaloids, tropane alkaloids, tea, herbal tea, culinary herbs, food toxins, food safety, LC-Q-ToF/MS, principal component analysis

## Abstract

Pyrrolizidine alkaloids are secondary metabolites produced by plants as a defense against insects. These can cause acute or chronic toxicity in humans. Therefore, avoiding potential poisoning from the consumption of tea and culinary plants contaminated with pyrrolizidine alkaloids (PAs), pyrrolizidine alkaloids N-oxides (PANOs), and tropane alkaloids (TAs) is important for human health and food safety. Therefore, it is important to determine the levels of these substances with reliable and highly accurate methods. In this study, the PAs, PANOs, and TAs in herbal teas and culinary herbs sold in Turkish markets were identified and their levels were determined. Thus, the general profiles of herbal teas and culinary herbs in Turkey were revealed, and the compliance of the total amounts of PA and TA with the regulations was examined. The identification and quantification of 25 PAs and N-oxides and 2 TAs (atropine and scopolamine) in the samples was performed with a liquid chromatography-quadrupole time-of-flight tandem mass spectrometer (LC-Q-ToF/MS). At least a few of these substances were detected in all of the tested herbal teas and culinary herbs. The total contents of the black tea, green tea, mixed tea, flavored tea, chamomile tea, sage tea, linden tea, fennel tea, rosehip tea, peppermint, and thyme samples ranged from 4.6 ng g^−1^ to 1054.5 ng g^−1^. The results obtained shed light on the importance of analyzing the total dehydro PA, PANO, and TA amounts in plant-based products consumed in diets with sensitive and accurate methods, and they highlight the necessity of performing these analyses routinely in terms of food safety.

## 1. Introduction

Pyrrolizidine alkaloids (PAs) are secondary plant metabolites that are naturally biosynthesized by angiosperms through defense mechanisms against herbivorous insects and pests. PAs and their N-oxides are a diverse class of secondary metabolites known as hepatotoxins in animals and humans. PAs are a class of phytotoxins that occur in an estimated 3% of flowering plants worldwide. More than 660 individual PAs and PANOs have been structurally characterized, and three plant families (Asteraceae, Boraginaceae, and Fabaceae) are among the most important sources of these toxins [1,2]. PA poisoning is caused by the consumption of herbal products containing these alkaloids for medicinal purposes or as culinary herbs. The pollution of plants with PAs, PANOs, and TAs, which are potentially harmful and toxic for humans and animals, can occur in different ways. Accidental or unintentional co-harvesting can be cited as the main source of plant contamination. Mixing the leaves of plants containing PAs with medicinal herbs or tea leaves and mixing the seeds of cereals with the seeds of plants containing PAs can be counted among other contamination pathways [3,4,5]. Beehive products (e.g., honey, royal jelly, and pollen food supplements) can naturally become contaminated with PAs through flowers, nectars, and pollen contents. It has been found that nectar from PA-diff or TA-producing plants is the main source of contamination of beehive products, and pollen from these flowers can additionally increase PA loads [6,7,8]. Animals may be fed with contaminated animal feed and plants. Thus, animal products (e.g., eggs, milk, cheese, and meat) can also be contaminated with Pas. Therefore, animal products may contain low levels of PAs [7,8,9,10]. In Hama and Strobel’s studies, it was reported that in regions with large populations of PA-producing plants, the surrounding soils and surface waters are vulnerable to PA pollution, and PAs can be transported from fields to surface waters by rain. With these studies, the importance of monitoring PAs in environmental samples has also been demonstrated [11,12].

Toxic PAs can cause liver cirrhosis and liver failure, pulmonary hypertension, cardiac hypertrophy, renal degeneration, carcinogenicity, and genotoxicity, which can be fatal [13,14,15,16,17]. Due to the nature of the plant materials, some teas may contain high amounts of PAs. In addition, teas can be contaminated with PAs from various plants (weeds) during growing and harvesting periods [18,19]. Medicinal preparations and teas can be important sources of human exposure to related pollutants as there may be poisonings and diseases caused by the consumption of medicinal preparations and teas prepared with PA-containing plants [19,20]. For livestock and human health reasons, as well as for food and feed safety, relevant PA/PANO and large PA/PANO quantities should be monitored comprehensively. Even in small amounts, they can cause toxic effects as a result of continuous exposure. Therefore, some of the European risk assessment bodies have made recommendations regarding daily intake to limit PA intake. The UK Committee on the Toxicity of Food Consumables and Environmental Chemicals and the German Federal Institute for Risk Assessment have recommended a maximum tolerable daily intake of 0.007 µg dehydro PA/kg body weight (bw). The Austrian Health and Food Safety agency does not allow any PA residues in final food products [21,22]. There is a need for regulations and standard methods for all countries for determining the allowable amounts of PAs/PANOs.

Tropane alkaloids (TAs) are an additional group of hazardous alkaloids that have been found to contaminate food and feed products. They are naturally occurring secondary metabolites of various plant families, including Brassicaceae, Solanaceae, and Erythroxylaceae. The most common alkaloids in TA-producing plants are naturally occurring (−)-hyoscyamine and (−)-scopolamine. Atropine, on the other hand, is a racemic mixture of (−)-hyoscyamine and (+)-hyoscyamine produced during the purification process of (−)-hyoscyamine. The wide distribution of TA-producing weeds in temperate and tropical regions worldwide can cause the occasional contamination of agricultural crops. It was reported by the EFSA CONTAM panel in 2013 that TAs have negative effects on human health, and they emphasized that the widespread TA-containing weeds in temperate and tropical regions may cause the accidental contamination of agricultural products as well as tea and herbal mixtures. Excessive anti-muscarinic activity in the central and autonomic nervous system are the main toxicological effects caused by atropine and scopolamine. Because of these effects on human health, the EFSA Panel has established a group acute reference dose of 0.016 μg/kg bw for the sum of the relevant TAs [23,24,25]. TAs, similar to PAs, can contaminate feeds, agricultural and animal products, and teas and herbal mixtures. Therefore, studies on TAs have investigated their presence in teas and herbal infusion matrices, as well as in honey and its products, cereal-based baby foods, other bee products, plants, cow’s milk, seeds, bread, and leafy vegetables [24,25,26,27,28,29,30,31,32]. LC-MS/MS systems or high-resolution mass systems were used in these studies.

According to the EFSA regulation, the pyrrolizidine alkaloid concentration is given as the sum of the PAs and PANOs [22]. According to the EC regulation, the maximum limit value for herbal infusions (dried products) is accepted to be 200 ug/kg. The limit value for herbal infusions of rooibos, anise, lemon balm, chamomile, thyme, mint, and lemon verbena as dried products and mixtures consisting of these dried products is only 400 ug/kg. The limit was determined to be 150 ug/kg for dried teas and flavored teas. The EC regulation suggested at least atropine and scopolamine as the tropane alkaloids to be analyzed, and their sum is given as the total TA concentration. The limit value for herbal infusions as dried products is 25 ug/kg [22,23,33,34].

Therefore, the safety management of PAs and TAs in teas and culinary herbs is necessary. Due to the worldwide widespread habit of consuming teas and other herbal infusions, if these are frequently contaminated, they can become the main source of human exposure to relevant contaminants. On the other hand, while studies on monitoring PAs in teas and culinary herbs in Turkey are very limited, according to literature reviews, no studies on TAs have been conducted [35,36,37,38].

The efficiency of PA and TA analyses depends on many factors, and they include the steps of extraction, separation, and identification. The analysis of PAs is mainly based on high performance liquid chromatography (HPLC), taking into account the physical and chemical properties of the PAs and TAs. Accurate and precise analytical methods using HPLC with mass spectrometry (MS) or tandem mass spectrometry MS/MS are commonly used in multicomponent analyses [12,39,40,41,42,43]. The use of LC-MS/MS or high-resolution mass spectrometry instruments is the dominant approach in the analysis of TAs. For the determination of TAs, SPE techniques are used prior to instrumental analysis [44,45,46,47,48,49,50].

Various purification methods such as thin layer chromatography, column chromatography, liquid-liquid extraction (LLE), and solid phase extraction (SPE) are applied to samples as pre-treatment methods. Among these, SPE is the most widely used, and it uses a strong cation exchange stationary phase based on the properties of the tertiary amine groups of PAs. To achieve the highest possible sensitivity, modern methods often rely on cleaning the samples by SPE prior to liquid chromatography-tandem mass spectrometry (LC-MS/MS) analysis. In general, the application of tandem mass spectrometry (MS/MS) results in low limits of detection (LOD), especially where multistage tandem mass spectrometry (MS3) or high resolution mass spectrometry systems are used [51,52,53,54,55,56,57,58].

In this study, the word “tea” refers to usable plant materials that are commercially called teas by the public or by the food industry, and “culinary herbs“ refers to plants used in the food industry and cooking. The main purpose of this study was to analyze the pyrrolizidine alkaloids and tropane alkaloids in different herbal teas and culinary herbs obtained from the Turkish market using a sensitive and high-accuracy LC-Q-ToF/MS system after sonification extraction with methanol. By evaluating the results obtained, the content of pyrrolizidine alkaloid was determined and its importance for human health and food safety was revealed. The distribution of PAs, PANOs, and TAs determined in Turkey is also explained by performing principal component analysis (PCA) according to the samples.

## 2. Materials and Methods

### 2.1. Chemicals and Reagents

Pure water from a Milli-Q water purification system (Millipore, Bedford, MA, USA) was used. Acetonitrile and methanol of LC-MS grade were purchased from Sigma-Aldrich (St. Louis, MO, USA). Formic acid eluent additive for the mobile phase was purchased from Merck (Darmstadt, Germany).

### 2.2. Samples

All of the tea samples (black tea (BT), green tea (GT), mixed tea (MT), and flavored tea (FT)), herbal tea samples (chamomile (CT), sage (ST), linden (LT), fennel (FT), and rosehips (RT)), and culinary herb samples (thyme (T) and peppermint (PM)) were obtained from Turkish supermarkets in the İzmir area and stored at room temperature until analysis.

### 2.3. Standards and Samples Preparation

A stock solution mixture was used where each of the standard compounds in methanol were in concentrations of 1.0 µg mL^−1^. Working solutions were prepared from this stock solution diluted with methanol. Working solutions were stored at −20 °C until analysis.

Different extractions were compared in the study by Avula et al. In this study, the extraction procedure, which mirrored that of Avula et al., obtained the best results, and it was applied [58]. Briefly, in the extraction method based on sonication with methanol, 50 mg of solid sample was sonicated in 2.5 mL of methanol for 30 min and then centrifuged at 3000 rpm for 15 min. The clear supernatant solution was transferred to a 10 mL metered flask. The procedure was repeated three more times, and the respective supernatants were combined. The final volume was adjusted to 10 mL with methanol and mixed well. Prior to injection, a sufficient volume (approximately 2 mL) was filtered through a 0.45 µm PTFE membrane filter and analyzed with the liquid chromatography hyphenated with quadrupole time of flight mass spectrometry (LC-Q-ToF/MS) system.

### 2.4. Instrumentation and Conditions

Chromatographic separation was performed using an Agilent HPLC 1260 Infinity system. The mass analysis was studied using an Agilent 6550 iFunnel high resolution accurate-mass Q-TOF/MS equipped with an Agilent Dual Jet Stream ElectroSpray Ionization (Dual AJS ESI) system according to the literature [58]. The interface operating in a positive ion mode was used for the analysis of the PAs (Agilent Technologies, Santa Clara, CA, USA). The acquisition was controlled by Agilent MassHunter Acquisition Software Ver. A.09.00 and the data were processed with MassHunter Qualitative Software Ver. B.07.00. The LC and MS detailed instrument conditions are given in Supplementary SM-1 and the MS/MS spectra with the product ions are given in Supplementary SM-2.

Although intermedine, intermedine-N-oxides, lycopsamine, lycopsamine-N-oxides, indicine, and indicine-N-oxides were present in the standard mixture, quantification was performed as the sum of these PAs and PANOs. Intermedine, lycopsamine and indicine have the same chemical formula (C_15_H_25_NO_5_), and the calculated [M+H]^+^ ion value was 300.1805. In the extracted ion chromatogram (EIC) of this ion, 2 peaks were observed at retention times of 9.029 min and 9.874 min. The N-oxides value of these substances (the (C_15_H_25_NO_6_) [M+H]^+^ ion) was 316.1760 and two peaks were observed at 15.169 min and 16.859 min in the EIC. Since the product ions seen in the MS/MS spectra were the same, the total peak areas could be determined in quantification. In addition, the mass resolutions calculated according to the MS/MS spectra of the two peaks were between 3099 and 5645. There was a need to study new methods for one-by-one calculations for intermedine, intermedine-N-oxides, lycopsamine, lycopsamine-N-oxides, indicine, and indicine -N-Oxides. Thus, it could be more clearly stated which of these PAs or PANOs were in the samples. All chromatograms and mass spectra are given in Supplementary SM-3 and SM-4 for these PAs and PANOs.

### 2.5. Principal Component Analysis

PCA was performed using MINITAB 15 Statistical Software. Similarities and differences between the main groups and the observations are presented as score plots. The loading plots were used to explain the relationships between the variables in the score plots and cluster observations [59,60]. All analysis results of the study are given in the Supplementary Materials (SM-5).

### 2.6. Method Validation

**Figure 1:**
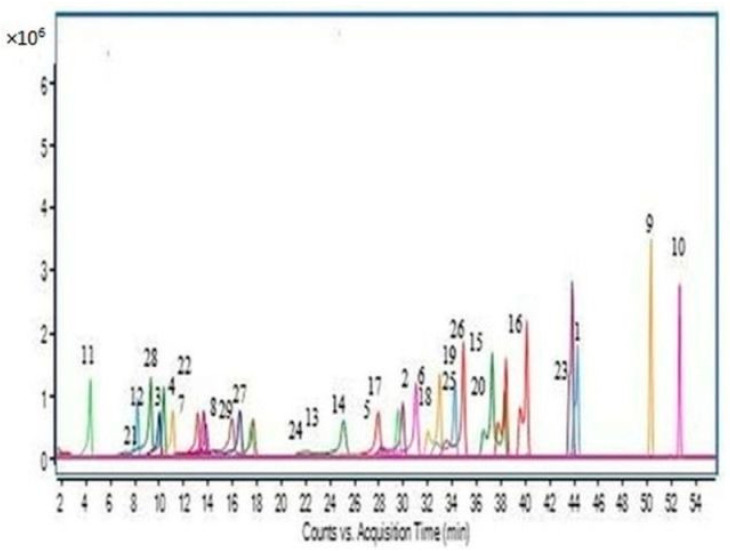
Extracted ion chromatogram of all the PA and TA standards. 1: Echimidine; 2: Erucifoline; 3: Europine; 4: Europine N-oxide; 5: Heliotrine; 6: Heliotrine N-oxide; 7: Jacobine; 8: Jacobine N-oxide; 9: Lasiocarpine; 10: Lasiocarpine N-oxide; 11: Monocrotaline; 12: Monocrotaline N-oxide; 13: Retrorsine; 14: Retrorsine N-oxide; 15: Senecionine; 16: Senecionine N-oxide; 17: Seneciphylline; 18: Seneciphylline N-oxide; 19: Senecivernine; 20: Senecivernine N-oxide; 21: Riddelline; 22: Riddelline N-oxide; 23: Senkirkine; 24: Trichodesmine; 25: Integerrimine; 26: Atropine; 27: Scopolamine; 28: Intermedine + Lycopsamine + Indicine; and 29: Intermedine-N-oxides + Lycopsamine-N-oxides + Indicine-N-oxides. Extracted ions of PAs and TAs are shown in different colors.

The LC-Q-ToF/MS method for the determination of the dehydro PAs, PANOs, and TAs in the teas, herbal teas, and culinary herbs were validated according to the SANCO/12571/2013 guideline [61]. The applied method for the determination of the dehydro PAs, PANOs, and TAs in the teas and herbal teas was validated in terms of linearity, recovery, repeatability, matrix effects, limit of detections (LODs), and limit of quantifications (LOQs). Quantification of the dehydro PAs, PANOs, and TAs was accomplished by external calibration. The calibration curves were determined at six different concentration levels ranging from 5.0 to 500.0 ng g^−1^ in methanol. Figure 1 shows a representative chromatogram of all the ions of the compounds. Recovery experiments were carried out in triplicate by spiking two different concentrations (5 and 20 ng/g of each PA, PANO, and TA compound) to blank herbal tea, peppermint, thyme, and chamomile samples. The repeatability of the method was expressed as the relative standard deviation (RSD) of the PA, PANO, and TA contents. LODs and LOQs were determined by the signal/noise ratio method. The calibration curves, LODs, LOQs, and relative standard deviation (RSD) values obtained for the teas and herbal teas for each compound are shown in Table 1. It was found that the linearity of the analytical response in the studied range was good and the correlation coefficients were appropriate.

Recovery (R) was calculated for each compound using the following formula:

R = (C_found_/C_added_) × 100, (1)

where C_found_ is the determined concentration of each compound after adding a known amount of standard solution to the blank sample and C_added_ is the known concentration of the compound solution added to the blank sample [62].

Table 1 provides the calibration data for the 27 reference PAs, PANOs, and TAs used in this study, and it includes the correct mass, mass error (diff), product ion, regression equation, correlation coefficient, detection limit, quantification limit, and RSD of repeatability.

According to the SANCO/12571/2013 guideline, the mean of the recovery limit values is accepted as 70–120% in the residue analysis [61]. It is seen in Table 2 that the recovery values of this study remained within these limits. In terms of the accuracy parameter, the method was valid for four different matrices (thyme, peppermint, tea, and chamomile) and all of the compounds (PAs, PANOs, and TAs).

For the estimation of the matrix effect, the calculation method in the study by Kwon et al. [63] was used. Linear calibration curves were calculated for each analyte using the peak areas for the matrix-matched standards and the solvent standards only. The matrix effect (ME) was calculated as follows: %ME = ((slope of matrix-matched calibration − slope of solvent-only calibration)/slope of solvent-only calibration) × 100 [63]. The matrix effect was investigated for four different matrices (thyme, mint, tea, and chamomile), and the results for each matrix are given in Table 2. The tables of the regression equations and the R^2^ values are given in the Supplementary Materials (Supplementary SM-6–SM-9) for the solvent and the matrix-matched calibration.

## 3. Results and Discussion

The data obtained on the total PAs and TAs were evaluated and their profiles were revealed by screening and measuring the herbal teas and culinary herbs consumed in Turkey. Table 3 summarizes the levels of the total dehydro PAs and TAs obtained for the various tea/herbal tea strains and edible herbs.

**Figure 2:**
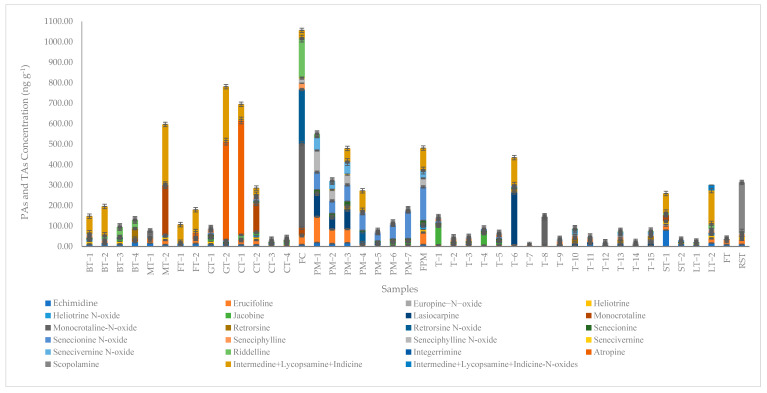
Distribution of the samples regarding their PA, PANO, and TA contents based on the 21 PAs and the 2 TAs.

According to the analysis results, one or more of the PAs and/or PANOs were found in all samples. TAs were found in only four of the herbal teas (green tea, chamomile tea, flavored tea, and rosehip tea). Echmidine, erucifoline, monocrotaline-n-oxide, and lasiocarpine were qualified and quantified in nearly all samples. When the samples were evaluated against the sum the intermedine, lycopsamine, and indicine-N-oxides, these PANOs were not found except for in the linden tea sample. The PA and TA levels and distribution are given in Table 3 and Figure 2.

Whether the total PAs exceeded the limit values was evaluated according to the EU regulations [33]. When the black, green, mixed, and flavored tea samples were examined, while the flavored teas remained below the 150 ng g^−1^ limit value, one sample from each of the other tea groups was above the limit value (black tea: 196.3 ng g^−1^, green tea: 781.6 ng g^−1^, and mixed tea: 596.2 ng g^−1^). According to the EU regulations, the limit value for chamomile, thyme, and peppermint dry products is 400 ng g^−1^ [33]. Only the fresh chamomile sample (1054.5 ng g^−1^) was well above the limit value. For the peppermint samples, three exceeded the limit value, with concentration values of 547.9, 479.1, and 480.8 ng g^−1^, respectively. With the exception of one of the thyme samples (435.9 ng g^−1^), the others remained below the limit value. The sage, linden, fennel, and rosehip teas all had concentrations below the 400 ng g^−1^ cutoff value for total pyrrolizidine alkaloid content. Scopolamine, one of the tropane alkaloids, was found in a mixed tea sample and in the rosehip tea in amounts of 35.1 ng g^−1^ and 244.5 ng g^−1^, respectively, while atropine was found in one flavored tea (29.3 ng g^−1^) and in the green tea (482.3 ng g^−1^) and chamomile tea (559.2 ng g^−1^) samples. The limit value for herbal infusions as dried products is above 25 ug/kg [34].

The black tea samples contained an average PA sum of 142.4 ng g^−1^. Atropine and scopolamine were not found in the black tea samples. The black tea samples all contained echminidine, lasiocarpine, and monocrotaline-N-oxide. The average concentration of the green tea samples was 434.5 ng g^−1^. Echminidine, lasiocarpine, monocrotaline-N-oxide, senecionine, and senecionine N-oxide were found in the samples, and 482.3 ng g^−1^ of atropine was found in the green tea sample. While the average was 336.3 ng g^−1^ in the mixed teas, echminidine and erucifoline are common PAs. Scopolamine was found in a sample at a value of 36.1 ng g^−1^. While echminidine and lasiocarpine are common in flavored teas, there was 29.3 ng g^−1^ of atropine found in one sample, and the PA average of the samples was 143.6 ng g^−1^. Unlike the other teas, monocrotaline was found in all chamomile tea samples, in addition to echminidine and erucifoline. While the echminidine, erucifoline, retrorsine, senecionine-N-oxide, riddelline, and atropine concentrations in the fresh chamomile were higher than in the other chamomile teas, scopolamine was found only in the fresh chamomile. The contents of the chamomile teas and the green tea were in alignment with the literature [21,64]. When the peppermint samples were examined, it was found that there was an average PA value of 308.4 ng g^−1^. TAs were not found in these samples. Erucifoline, lasiocarpine, senecionine, senecionine N-oxide, and seneciphylline-N-oxides were found in all samples. The total contents of the freshly analyzed chamomile and peppermint samples were higher than those of the dried chamomile and peppermint samples. Particularly because peppermint is used in salads and beverages, it is more important to control its contents for human health. Unlike the other teas, the sage and linden teas contained heliotrine, and the linden tea also contained heliotrine-N-oxide. Apart from this, there was 244.7 ng g^−1^ of scopolamine in the rosehip tea sample, and this type of tea is consumed quite a lot during the winter months. The fact that the numbers and types of PAs found in the herbal tea samples were different was in line with the literature [65,66,67,68,69]. The thyme samples contained the most lasiocarpine, monocrotaline-N-oxides, retrorsine, senesiphylline, and senesiphylline-line-N-oxides, with an average of 87.8 ng g^−1^. TAs were not found in the thyme samples. When the samples were examined in terms of the sum of their intermedin, lycopsamine, and indicin contents, which were found in most of the tea samples, they were detected in only one of the thyme samples (137.5 ng g^−1^). The sum of the intermedin, lycopsamine, and indicin contents could not be measured in the fennel and rosehip teas because it was below the LOQ value. When the total of the intermedin, lycopsamine, and indicin N oxides contents were examined, only one linden tea contained a value of 27.1 ng g^−1^. The wide PA content variations within the same species could be explained by the fact that the samples came from different plant varieties and, consequently, they were subjected to different growing, harvesting, storage, and transportation conditions.

**Figure 3:**
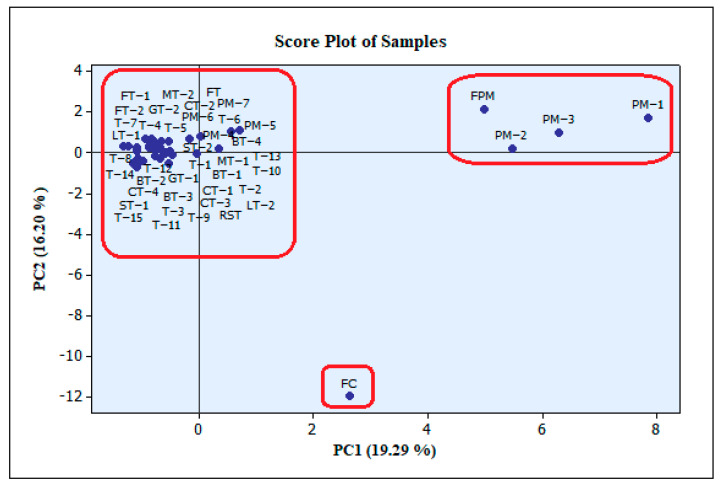
Score plot of the PCA results for all samples.

**Figure 4:**
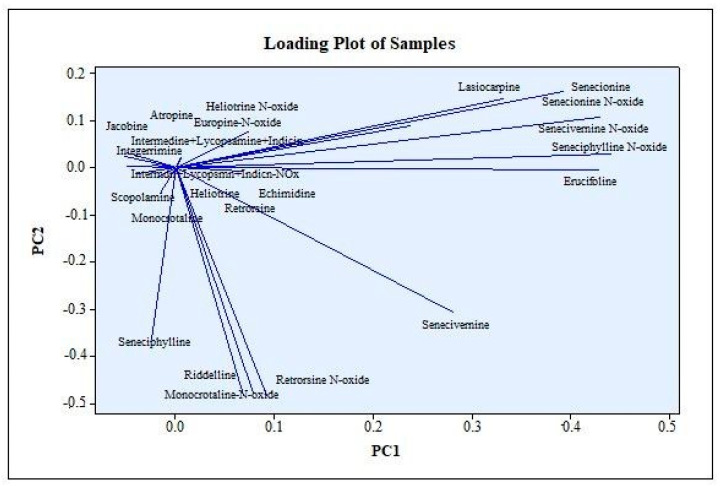
Loading plot of the PCA results for all samples.

After determining the amounts of the PAs, PANOs, and TAs in the samples, PCA analysis was performed to more clearly reveal the similarities and differences in the contents. The PCA scores and loading plots can be seen in Figure 3. First, general information was obtained by PCA using the whole dataset (Figure 3 and Figure 4). The PCA analysis for the thyme, tea, peppermint, and chamomile samples was performed for each sample group separately (Supplementary SM-10–13). The columns with zero values were not included in the analysis.

Although the overall effect of PC1 and PC2 was 35.49%, the separate classifications of the fresh chamomile and fresh mint samples is clearly seen in Figure 3. As can be seen from the PCA score plot, the fresh peppermint and dry peppermint samples were separated from the other samples and showed similar characteristics within themselves (Figure 3). The fresh chamomile sample was separated from all other samples. The grouping of fresh chamomile separately from the other teas was realized under the influence of retrorsine-N-oxide, seneciphylline, riddelline, senecivernine, and monocrotaline-N-oxides. While the peppermint samples differed from the other samples, they also showed similarity with the fresh peppermint sample. The fresh peppermint was separated by the effects of erucifoline, senecionine, lasiocarpine, seneciphylline-N-oxide, senecivernine-N-oxide, and senecionine N-oxide. It could be said that the effect of the PANOs was effective for the separation of the fresh plant samples from the other dry samples, and this may have indicated that the differences in the contents of the teas and culinary herbs may also have been due to the drying process. The differences between the fresh samples and the dry samples may also have revealed the importance of the drying process because the total PA amounts in the fresh peppermint and the fresh chamomile samples were above the limit values.

In this study—the first of its kind—conducted to determine the total PA and TA amounts for some herbal tea and edible herb samples in Turkey, it was determined that some samples were above the limit values. Therefore, the continued quality control of herbal teas, edible plants, herbs, and other foodstuffs that may be contaminated with PAs is important.

## 4. Conclusions

Herbal teas and culinary herbs are products that are constantly consumed in daily diets. Although these teas and culinary herbs have therapeutic benefits, they can also pose potential health risks due to the toxic substances they contain. Therefore, PAs, PANOs, and TAs should be monitored with sensitive and accurate analytical techniques in terms of human health and food safety. In order to determine their total amounts, extraction techniques should be developed quickly to be cost-effective and suitable for routine analysis, and all structures should be defined and measured by instrumental methods. In this study, it was determined that it is important to control these products as the total PA and TA contents in some of the teas and culinary herb samples were present in amounts that exceeded the legal limits, and the PAs were screened and quantified in all samples. Thus, it needs to be ensured that only products complying with legal regulations reach consumers. In addition, the importance of applying regulations for the determination of PA and TA contents in teas and culinary herbs and continuous data collection on the presence of alkaloids in these products has been demonstrated. This study also sheds light on the optimization of methods that can be applied to other food products and feeds that may be contaminated with PAs, PANOs, and TAs.

## Figures and Tables

**Table 1:** Retention time, accurate mass data, mass error, calibration data, LOD, LOQ, and repeatability for the 25 PAs and PANOs and the two TA standards determined using LC-Q-ToF-MS.

#	Compound Name	RT ^a^ (min)	Formula	Mass	[M+H]^+^ Calculated	[M+H]^+^ Experimental	Product Ion	Mass Error (Diff) (ppm)	Regression Equation	R^2^	LOD ^a^ (ng g^−1^)	LOQ ^a^ (ng g^−1^)	Repeatability RSD%
1	Echimidine	43.88 ± 0.02	C_20_H_31_NO_7_	397.2101	398.2173	398.2175	120.0808	0.50	y = 113,975x − 188,691	0.9996	0.163 ± 0.004	0.550 ± 0.010	4.11
2	Erucifoline	31.65 ± 0.02	C_18_H_23_NO_6_	349.1525	350.1604	350.1595	120.0807	−2.57	y = 98,243x − 438,608	0.9997	0.424 ± 0.004	1.417 ± 0.009	3.49
3	Europine	8.73 ± 0.02	C_16_H_27_NO_6_	329.1838	330.1911	330.1919	138.0913	2.42	y = 75,299x + 40,588	0.9998	0.520 ± 0.003	1.730 ± 0.012	1.06
4	Europine N-oxide	10.58 ± 0.01	C_16_H_27_NO_7_	345.1788	346.186	346.1857	172.0973	−0.87	y = 75,711x + 39,711	0.9999	0.782 ± 0.005	2.609 ± 0.015	1.32
5	Heliotrine	26.07 ± 0.02	C_16_H_27_NO_5_	313.1889	314.1962	314.1964	138.0916	0.64	y = 92,882x − 259,552	0.9994	0.793 ± 0.008	2.646 ± 0.025	3.92
6	Heliotrine N-oxide	30.48 ± 0.01	C_16_H_27_NO_6_	329.1838	330.1911	330.1911	172.0971	0.00	y = 116,893x − 192,370	0.9995	0.363 ± 0.007	1.207 ± 0.026	3.24
7	Jacobine	10.58 ± 0.01	C_18_H_25_NO_6_	351.1682	352.1760	352.1751	120.0807	−2.56	y = 83,888x − 206,710	0.9994	0.867 ± 0.008	2.890 ± 0.024	0.39
8	Jacobine N-oxide	14.08 ± 0.01	C_18_H_25_NO_7_	367.1631	368.1709	368.1702	296.1490	−1.90	y = 49,715x + 220,560	0.9992	0.750 ± 0.003	2.501 ± 0.008	0.44
9	Lasiocarpine	49.62 ± 0.01	C_21_H_33_NO_7_	411.2257	412.233	412.2325	120.0809	−1.21	y = 228,585x − 70,155	0.9997	0.141 ± 0.002	0.472 ± 0.004	4.18
10	Lasiocarpine N-oxide	52.10 ± 0.01	C_21_H_33_NO_8_	427.2206	428.2279	428.228	254.1385	0.23	y = 180,097x + 785,864	0.9992	0.105 ± 0.006	0.357 ± 0.015	2.89
11	Monocrotaline	3.60 ± 0.02	C_16_H_23_NO_6_	325.1525	326.1598	326.1593	121.0885	−1.53	y = 84,062x + 247,397	0.9998	0.306 ± 0.006	1.022 ± 0.016	2.07
12	Monocrotaline N-oxide	7.71 ± 0.02	C_16_H_23_NO_7_	341.1475	342.1547	342.1546	137.0834	−0.29	y = 49,678x − 16,030	0.9999	0.490 ± 0.004	1.640 ± 0.015	1.49
13	Retrorsine	22.92 ± 0.01	C_18_H_25_NO_6_	351.1682	352.1755	352.1754	120.0808	−0.28	y = 66,827x − 38,398	0.9996	0.665 ± 0.006	2.211 ± 0.030	4.18
14	Retrorsine N-oxide	25.06 ± 0.01	C_18_H_25_NO_7_	367.1631	368.1704	368.1698	118.0649	−1.63	y = 43,255x + 203,752	0.9997	0.778 ± 0.009	2.593 ± 0.031	2.86
15	Senecionine	35.20 ± 0.01	C_18_H_25_NO_5_	335.1733	336.1805	336.1814	120.0808	2.68	y = 86,527x − 112,901	0.9998	0.564 ± 0.010	1.882 ± 0.033	3.05
16	Senecionine N-oxide	38.41 ± 0.02	C_18_H_25_NO_6_	351.1682	352.1755	352.1762	118.0650	1.99	y = 14,0271x − 217,910	0.9997	0.230 ± 0.004	0.771 ± 0.015	4.45
17	Seneciphylline	27.48 ± 0.01	C_18_H_23_NO_5_	333.1576	334.1649	334.1648	120.0807	−0.30	y = 130,884x − 275,878	0.9995	0.394 ± 0.010	1.318 ± 0.032	1.32
18	Seneciphylline N-oxide	31.65 ± 0.01	C_18_H_23_NO_6_	349.1525	350.1598	350.1598	120.0807	0.00	y = 103,691x − 152,094	0.9997	0.382 ± 0.007	1.270 ± 0.023	1.15
19	Senecivernine	33.51 ± 0.02	C_18_H_25_NO_5_	335.1733	336.1811	336.1803	120.0808	−2.38	y = 157,349x + 277,937	0.9998	0.552 ± 0.010	1.838 ± 0.032	4.75
20	Senecivernine N-oxide	36.44 ± 0.01	C_18_H_25_NO_6_	351.1682	352.1760	352.1760	118.0651	0.00	y = 142,711x + 375,742	0.9997	0.415 ± 0.006	1.387 ± 0.015	3.19
21	Riddelline	6.42 ± 0.01	C_18_H_23_NO_6_	349.1525	350.1598	350.1600	120.0807	0.57	y = 75,303x + 122,694	0.9998	0.342 ± 0.006	1.144 ± 0.019	1.98
22	Riddelline N-oxide	10.53 ± 0.02	C_18_H_23_NO_7_	365.1475	366.1547	366.1545	119.0728	−0.55	y = 56,263x + 372,724	0.9995	0.824 ± 0.006	2.743 ± 0.025	0.62
23	Senkirkine	42.02 ± 0.01	C_19_H_27_NO_6_	365.1838	366.1911	366.1911	168.1025	0.00	y = 200,533x + 439,894	0.9998	0.767 ± 0.008	2.558 ± 0.023	2.49
24	Trichodesmine	20.67 ± 0.02	C_18_H_27_NO_6_	353.1838	354.1911	354.1912	222.1495	0.28	y = 121,840x + 173,831	0.9997	0.845 ± 0.004	2.817 ± 0.015	1.69
25	Integerrimine	34.61 ± 0.01	C_18_H_25_NO_5_	335.1733	336.1805	336.1803	120.0807	−0.59	y = 100,282x + 203,926	0.9996	0.462 ± 0.005	1.538 ± 0.016	3.21
26	Atropine	34.64 ± 0.01	C_17_H_23_NO_3_	289.1678	290.1756	290.1747	124.1120	−3.10	y = 136,867x + 284,758	0.9994	0.160 ± 0.005	0.540 ± 0.017	3.89
27	Scopolamine	16.13 ± 0.02	C_17_H_21_NO_4_	303.1471	304.1549	304.1538	138.0912	−3.62	y = 66,561x − 284,524	0.9990	0.181 ± 0.005	0.599 ± 0.018	1.27
28 ^b^	Intermedine Lycopsamine Indicine	9.03 ± 0.01 9.07 ± 0.01	C_15_H_25_NO_5_	299.1733	300.1811	300.1807	94.0648	−1.33	y = 200,383x − 216,672	0.9991	0.681 ± 0.007 0.723 ± 0.008	2.267 ± 0.024 2.411 ± 0.025	2.30 2.28
29 ^b^	Intermedine-N-oxides Lycopsamine-N-oxides Indicine-N-oxides	15.17 ± 0.01 16.86 ± 0.01	C_15_H_25_NO_6_	315.1681	316.1760	316.1759	172.0972	−0.32	y = 141,661x + 113,442	0.9998	0.754 ± 0.011 0.840 ± 0.008	2.518 ± 0.037 2.800 ± 0.027	1.12 1.43

^a^, mean ± SD; ^b^, peaks 28 and 29 represent the sum of intermedin, lycopsamine, and indicine and the N-oxides, respectively.

**Table 2:** Recovery and matrix effect for the 27 reference PAs, PANOs, and TAs used in this study for thyme, tea, peppermint, and chamomile (n = 3).

Compound Name	R (%) Thyme 5 ng g^−1^	R (%) Tea 5 ng g^−1^	R (%) Peppermint 5 ng g^−1^	R (%) Chamomile 5 ng g^−1^	R (%) Thyme 20 ng g^−1^	R (%) Tea 20 ng g^−1^	R (%) Peppermint 20 ng g^−1^	R (%) Chamomile 20 ng g^−1^	ME % Thyme	ME % Tea	ME % Peppermint	ME % Chamomile
Echimidine	92 ± 2	101 ± 3	101 ± 2	94 ± 2	103 ± 1	99 ± 2	99 ± 2	103 ± 1	14.1	2.3	−5.6	5.5
Erucifoline	94 ± 3	91 ± 2	102 ± 2	105 ± 1	94 ± 3	99 ± 2	96 ± 1	100 ± 2	−4.0	−6.7	−1.1	−0.6
Europine	102 ± 2	101 ± 2	102 ± 1	101 ± 2	90 ± 2	90 ± 1	89 ± 2	93 ± 1	8.7	11.9	−2.0	1.7
Europine N-oxide	104 ± 1	106 ± 2	103 ± 2	99 ± 2	99 ± 2	96 ± 2	92 ± 2	95 ± 1	−3.9	3.2	−1.8	8.6
Heliotrine	106 ± 2	100 ± 2	103 ± 1	109 ± 2	101 ± 1	95 ± 2	97 ± 2	94 ± 3	−2.2	3.8	−1.1	1.3
Heliotrine N-oxide	97 ± 2	97 ± 2	103 ± 1	103 ± 2	102 ± 1	103 ± 1	95 ± 1	96 ± 3	−9.5	−10.0	−7.2	−3.7
Jacobine	97 ± 1	90 ± 1	90 ± 2	99 ± 2	91 ± 2	79 ± 2	99 ± 2	95 ± 2	−11.8	−1.2	−3.1	−2.1
Jacobine N-oxide	95 ± 3	104 ± 2	113 ± 2	103 ± 1	89 ± 1	99 ± 2	101 ± 1	97 ± 2	2.8	−10.5	2.1	−1.9
Lasiocarpine	103 ± 1	105 ± 2	104 ± 2	104 ± 2	97 ± 2	103 ± 1	100 ± 2	96 ± 2	−1.9	−8.4	−5.9	−2.0
Lasiocarpine N-oxide	101 ± 2	99 ± 1	94 ± 1	101 ± 2	103 ± 2	102 ± 2	100 ± 2	104 ± 2	10.6	−3.0	−2.9	7.1
Monocrotaline	100 ± 2	98 ± 2	101 ± 2	88 ± 2	104 ± 2	94 ± 2	96 ± 2	88 ± 2	4.1	−16.2	−0.8	−0.9
Monocrotaline N-oxide	98 ± 2	101 ± 2	87 ± 2	93 ± 1	99 ± 1	97 ± 2	97 ± 2	91 ± 2	−1.7	−12.8	2.2	−2.4
Retrorsine	93 ± 1	86 ± 2	89 ± 2	94 ± 2	98 ± 2	95 ± 2	99 ± 1	95 ± 3	−1.7	−12.0	0.5	−0.9
Retrorsine N-oxide	99 ± 2	89 ± 2	88 ± 2	95 ± 2	98 ± 2	99 ± 2	98 ± 3	97 ± 2	9.8	−15.6	2.9	4.3
Senecionine	92 ± 2	97 ± 2	76 ± 2	92 ± 3	99 ± 2	98 ± 2	97 ± 2	101 ± 1	4.9	10.6	−1.5	2.0
Senecionine N-oxide	99 ± 2	101 ± 2	103 ± 2	104 ± 2	102 ± 2	87 ± 2	102 ± 2	99 ± 2	3.6	−11.9	−4.4	1.4
Seneciphylline	96 ± 3	94 ± 2	103 ± 2	97 ± 1	95 ± 2	87 ± 2	96 ± 2	98 ± 3	−14.8	−16.1	−1.8	0.9
Seneciphylline N-oxide	100 ± 1	99 ± 1	97 ± 2	96 ± 2	100 ± 3	94 ± 2	94 ± 2	96 ± 2	4.6	−7.6	−3.6	−3.4
Senecivernine	98 ± 3	99 ± 2	97 ± 1	97 ± 2	102 ± 2	77 ± 1	89 ± 1	99 ± 2	−12.9	−16.8	−8.3	−12.8
Senecivernine N-oxide	99 ± 2	98 ± 2	89 ± 2	97 ± 3	101 ± 2	100 ± 2	95 ± 2	99 ± 2	1.6	−13.3	−4.6	−7.6
Riddelline	99 ± 1	95 ± 2	102 ± 2	104 ± 2	95 ± 2	78 ± 2	93 ± 3	93 ± 1	−2.4	−12.5	−1.6	1.1
Riddelline N-oxide	98 ± 2	99 ± 3	103 ± 2	83 ± 1	91 ± 1	75 ± 2	81 ± 2	89 ± 2	−5.4	−14.8	3.1	2.9
Senkirkine	85 ± 2	90 ± 2	89 ± 2	96 ± 2	92 ± 2	81 ± 2	99 ± 2	102 ± 2	4.7	−8.5	−1.9	1.3
Trichodesmine	100 ± 2	95 ± 2	99 ± 2	98 ± 2	102 ± 1	86 ± 3	90 ± 2	96 ± 2	1.4	−12.1	−2.3	−6.1
Integerrimine	99 ± 2	98 ± 3	98 ± 2	93 ± 2	90 ± 2	91 ± 4	89 ± 2	89 ± 2	11.6	4.8	1.1	13.1
Atropine	88 ± 2	80 ± 2	83 ± 2	86 ± 2	100 ± 2	79 ± 2	91 ± 3	100 ± 2	−4.6	−11.12	10.4	6.4
Scopolamine	90 ± 2	80 ± 2	79 ± 2	87 ± 1	86 ± 2	83 ± 2	88 ± 2	94 ± 1	2.5	−4.2	0.8	4.4
Intermedine Lycopsamine Indicine	101 ± 2	94 ±3	100 ± 2	100 ± 1	102 ± 1	98 ± 2	95 ± 3	99 ± 3	−10.3	9.7	3.5	−7.0
Intermedine-N-oxides Lycopsamine-N-oxides Indicine-N-oxides	97 ± 1	95 ± 3	103 ± 1	102 ± 2	98 ± 2	99 ± 3	98 ± 1	100 ± 2	−12.0	−9.4	−6.4	−5.6

**Table 3:** PA and TA levels in analyzed the tea and culinary herb samples.

Sample	Number of Samples	Mean of the PAs	Concentration Range of the PAs (ng g^−1^)	Mean of the TAs	Concentration Range of the TAs (ng g^−1^)	Number of PAs + TAs
Black tea	4	142.4	95.2–196.3	<LOQ	<LOQ	8
Green tea	2	434.5	87.4–781.6	241.1	<LOQ−482.3	7
Mixed tea	2	336.3	76.3–596.3	17.5	<LOQ−35.1	6
Flavored tea	2	143.6	107.4–179.8	14.6	<LOQ−29.3	5
Chamomile tea	5	421.1	31.5–1054.5	111.8	<LOQ−559.2	10
Peppermint Tea	8	308.4	73.6–547.9	<LOQ	<LOQ	10
Thyme	15	87.8	4.6–435.9	<LOQ	<LOQ	6
Sage tea	2	144.9	30.8–259.1	<LOQ	<LOQ	8
Linden tea	2	160.9	21.1–300.8	<LOQ	<LOQ	8
Fennel tea	1	-	35.7	-	<LOQ	4
Rosehip tea	1	-	68.6	-	244.7	8

## Data Availability

The data can be requested from the author if needed.

## Supplementary Materials

The following supporting information can be downloaded at https://www.mdpi.com/article/10.3390/foods12193572/s1, SM-1, the LC-Q-ToF/MS method; SM-2, the MS/MS spectra for the PAs, PANOs, and TAs; SM-3, the MS EIC, MS/MS EIC, and mass spectrum of the product ions of intermedine, lycopsamine, and indicine; SM-4, the MS EIC, MS/MS EIC, and mass spectrum of the product ions of intermedine-N-oxides, lycopsamine-N-oxides, and indicine -N-oxides; SM-5, the data for the PAs and Tas in the samples; SM-6, the regression equations and R^2^ values of the solvent and matrix-matched calibration for the thyme matrix; SM-7, the regression equations and R^2^ values for the solvent and matrix-matched calibration for the tea matrix; SM-8, the regression equations and R^2^ values for the solvent and matrix-matched calibration for the chamomile matrix; SM-9, the regression equations and R^2^ values for the solvent and matrix-matched calibration for the peppermint matrix; SM-10, the scores and loading plots of the thyme samples; SM-11, the scores and loading plots of the tea samples; SM-12, the scores and loading plots of the chamomile samples; and SM-13, the scores and loading plots of the peppermint samples.
