# Supplementary material for: Assessment of Toxic Pyrrolizidine and Tropane Alkaloids in Herbal Teas and Culinary Herbs Using LC-Q-ToF/MS
Source: Foods. 2023 Sep 26;12(19):3572. doi: 10.3390/foods12193572 (PMC10572649; doi:10.3390/foods12193572)

Supplementary Materials

Assessment of Toxic Pyrrolizidine and Tropane Alkaloids in Herbal Teas and Culinary Herbs Using LC-Q-ToF/MS

Zinar Pinar Gumus

Central Research Test and Analysis Laboratory Application and Research Center (EGE-MATAL), Ege University, 35100 Izmir, Turkey; zinar.pinar.gumus@ege.edu.tr

SM-1. LC-Q-ToF/MS Method

The liquid chromatographic system was an Agilent Series 1260 comprised of the following modular components: binary pump, a vacuum solvent degasser, an autosampler, and a thermostatically controlled column compartment (Agilent Technologies, Santa Clara, CA, USA). Separation was achieved on an Agilent Poroshell 120 EC-C18 (2.1 x 150 mm, 2.7 µm) column. The mobile phase consisted of water with 0.1% formic acid(A) and acetonitrile with 0.1% formic acid (B) at a flow rate of 0.27 mL/min, with the following gradient scheme: 0–23 min, 3– 4% B; 23–45 min, 4–15% B; 45–55 min, 15–25% B and in next 2 min to 100% B. Each run was followed by a 3 min wash with 100% B and an equilibration period of 5 min with 3% B. Ten microliters of sample was injected. The column temperature was set at 40 °C. The mass spectrometric analysis was performed with a QToF-MS/MS (6550 iFunnel, Agilent Technologies, Santa Clara, CA, USA) equipped with an ESI source with Jet Stream technology using the following parameters: drying gas (N_2) flow rate, 11.0 L/min; drying gas temperature, 300 °C; nebulizer, 35 psig, sheath gas temperature, 325 °C; sheath gas flow, 10 L/min; capillary, 3500 V; skimmer, 65 V; Oct RF V, 750 V; fragmentor voltage, 125 V. MS/MS spectra were collected with collision energies of 30 eV. Each sample was analyzed in positive mode over the range of m/z 50–950. Accurate mass measurements were obtained by means of reference ion correction using reference masses at m/z 121.0509 and 922.0098 in positive ion mode.

SM-2. The MS/MS spectra for each Pas, PANOs, and TAs.

Echimidine

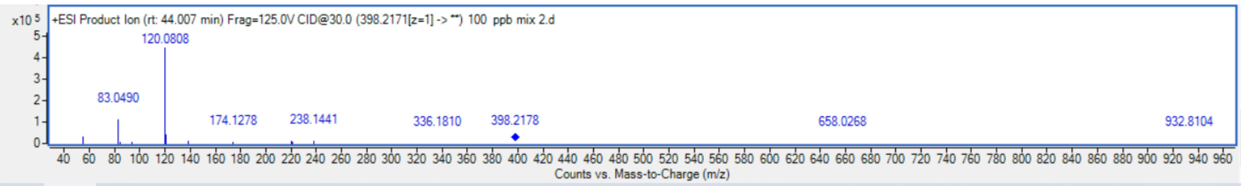

Erucifoline

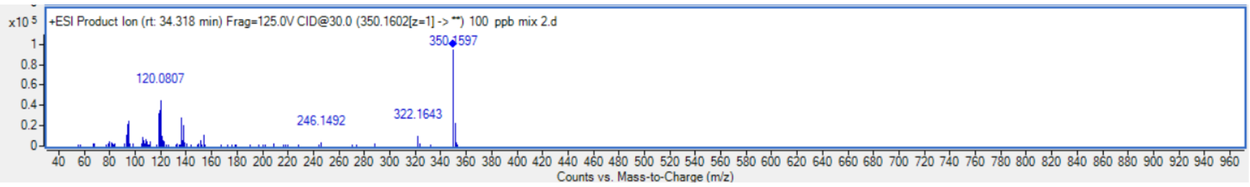

Europine

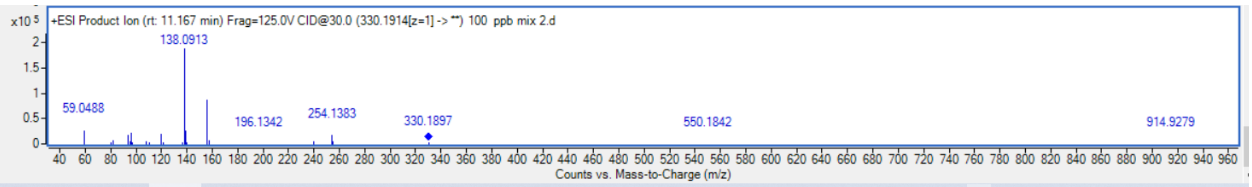

Europine-N-oxide

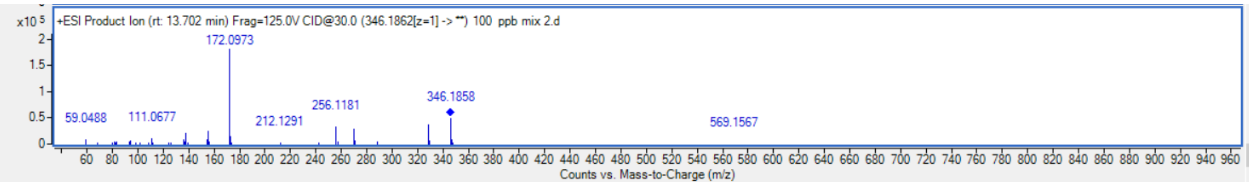

Heliotrine

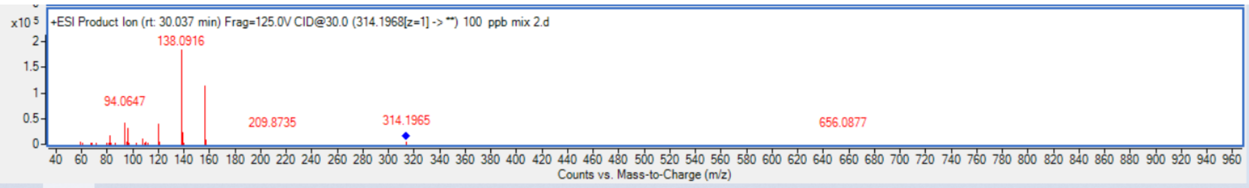

Heliotrine N-oxide

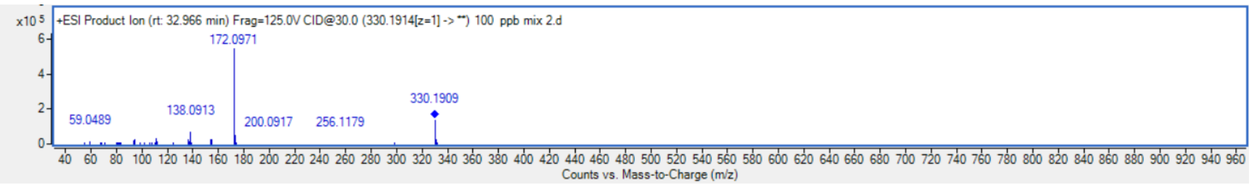

Jacobine

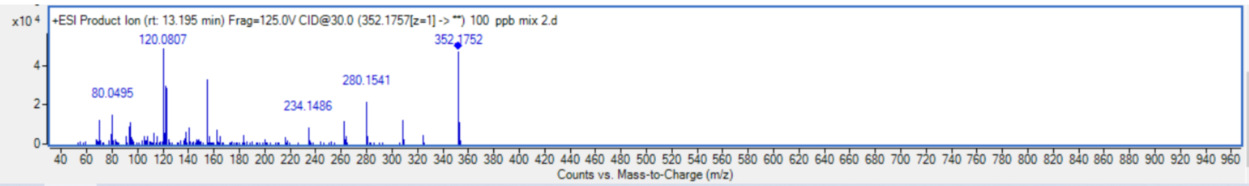

Jacobine N-oxide

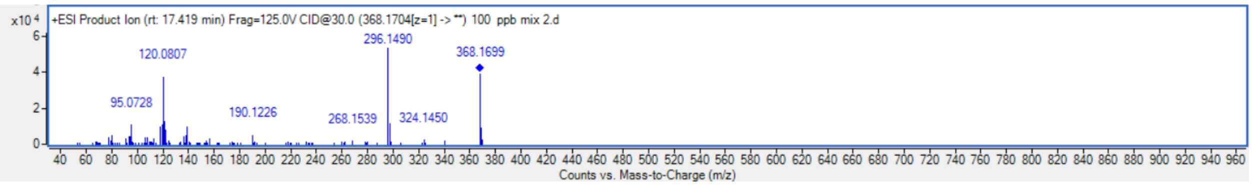

Lasiocarpine

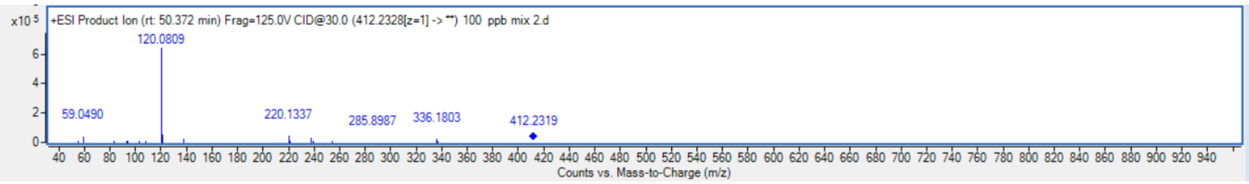

Lasiocarpine N-oxide

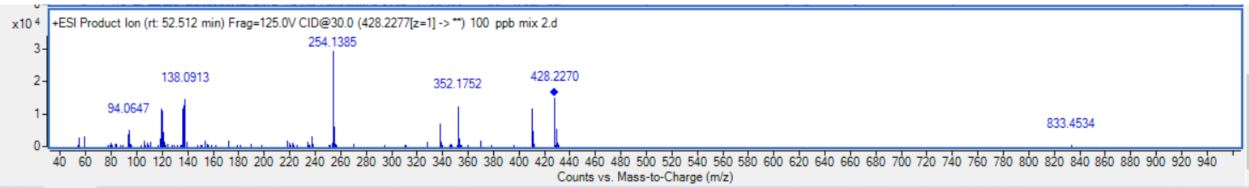

Monocrotaline

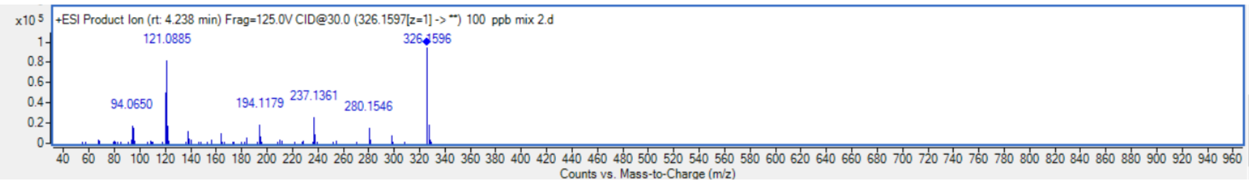

Monocrotaline-N-oxide

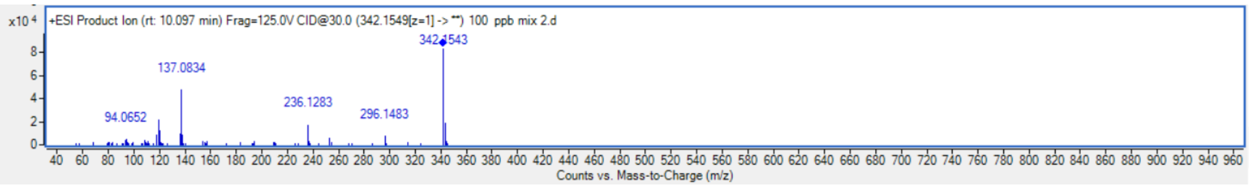

Retrorsine

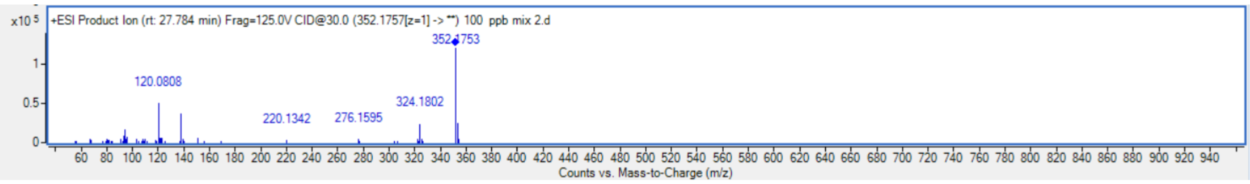

Retrorsine N-oxide

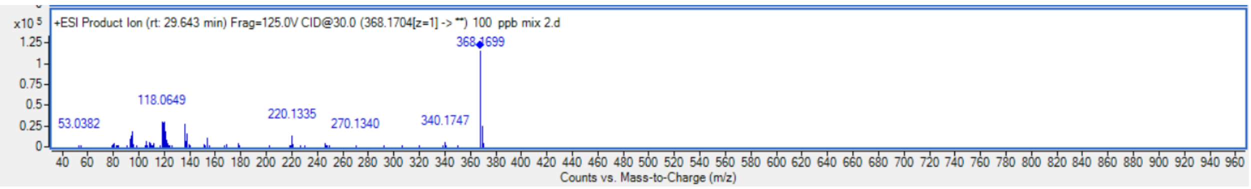

Senecionine

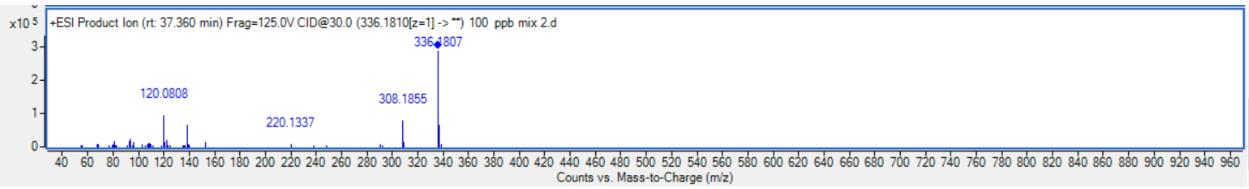

Senecionine N-oxide

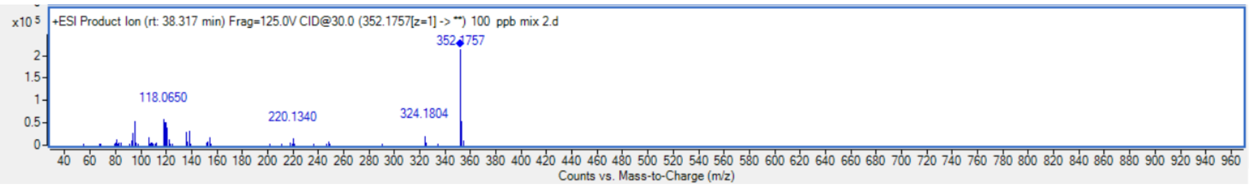

Seneciphylline

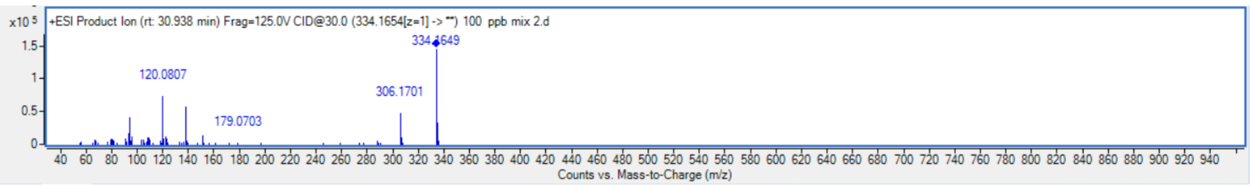

Seneciphylline N-oxide

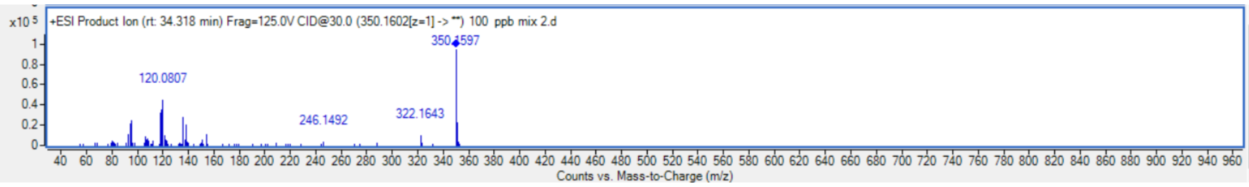

Senecivernine

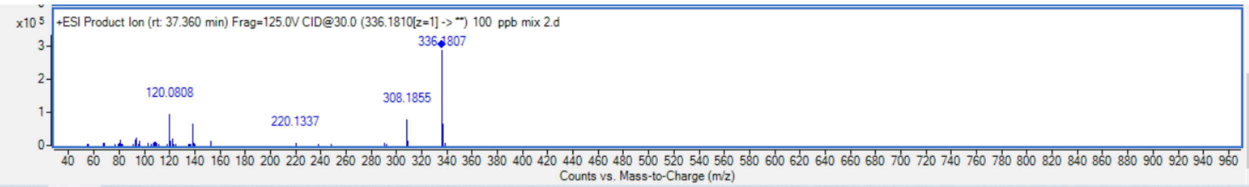

Senecivernine N-oxide

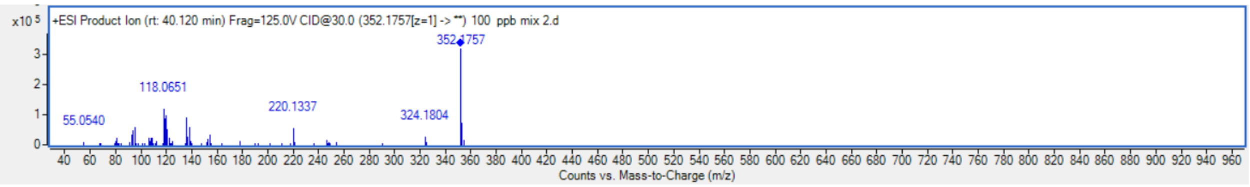

Riddelline

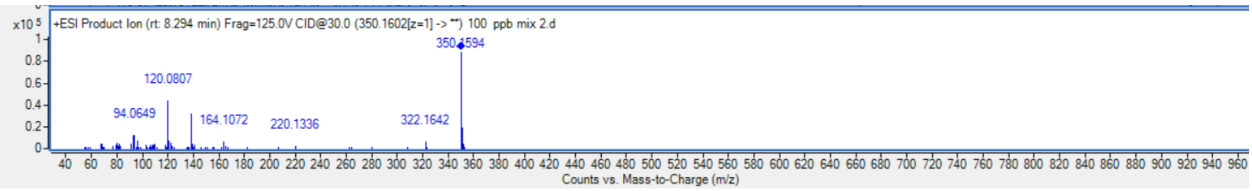

Riddelline *N-oxide*

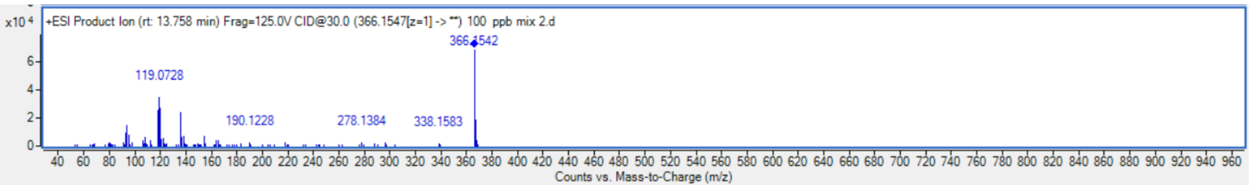

Senkirkine

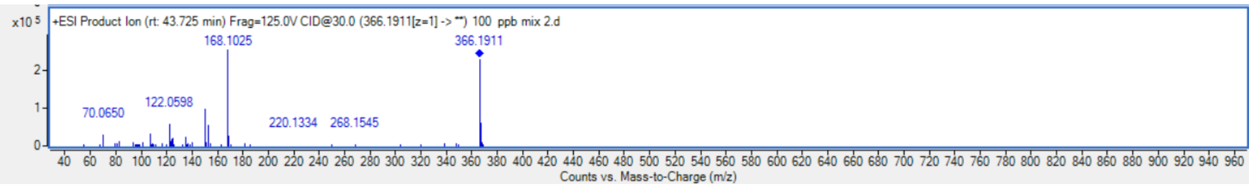

Trichodesmine

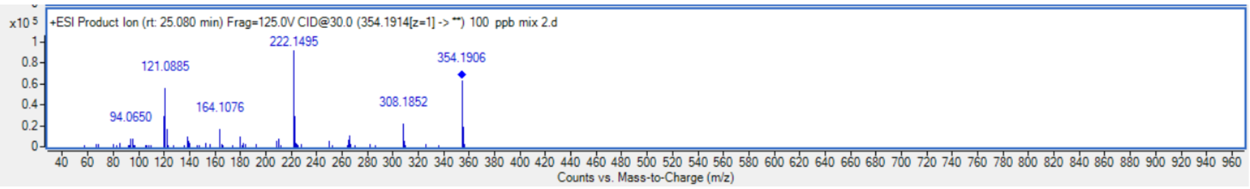

Integerrimine

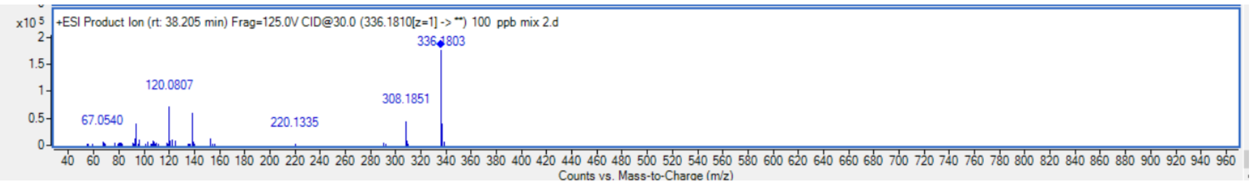

Atropin

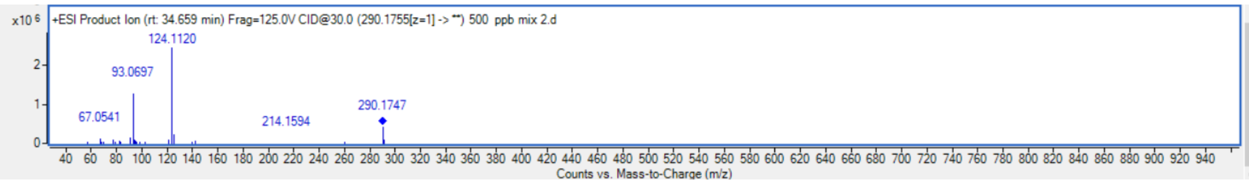

Scopolamine

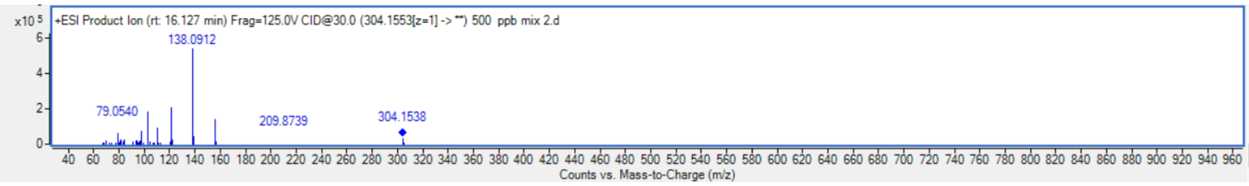

SM-3. MS EIC, MS/MS EIC, mass spectrum of product ion of Intermedine, Lycopsamine, Indicine

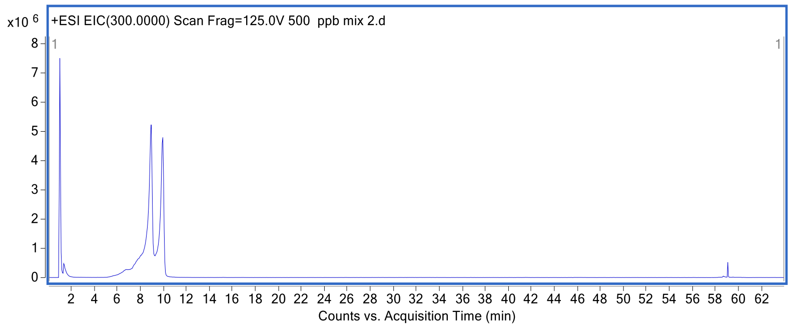

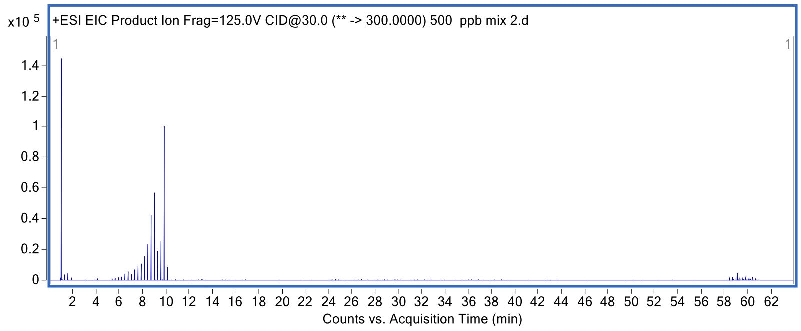

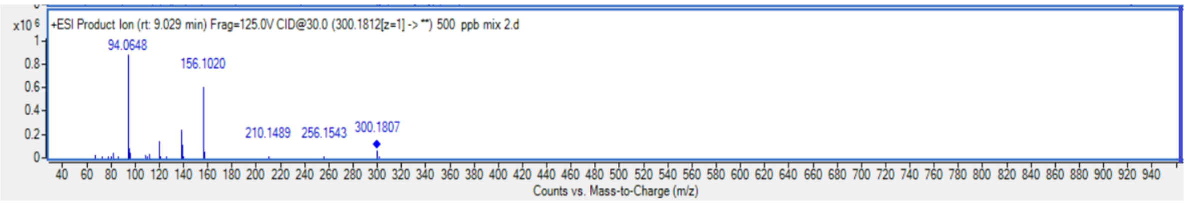

SM-4. MS EIC, MS/MS EIC, mass spectrum of product ion of Intermedine-N-oxides, Lycopsamine-N-oxides, Indicine -N-Oxides

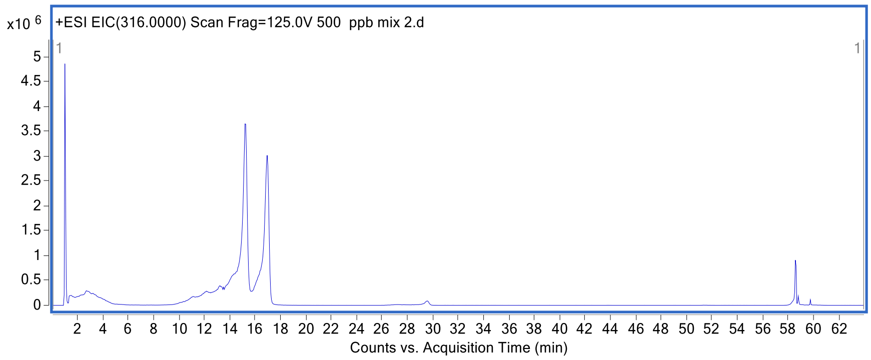

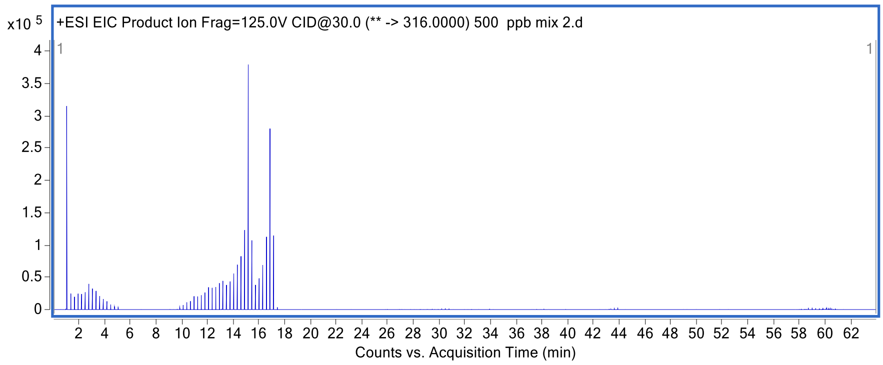

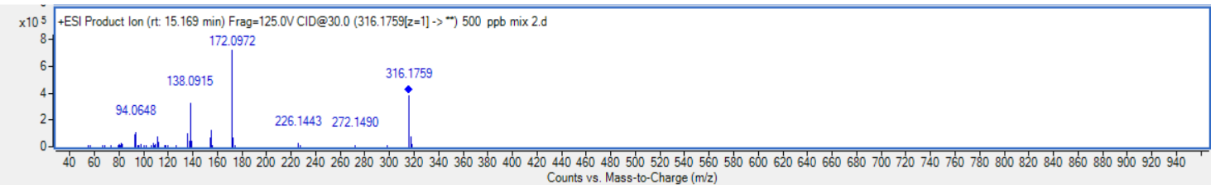

SM. 5. Data of samples for PAs and TAs

Compound	BT-1	BT-2	BT-3	BT-4	MT-1	MT-2	FT-1	FT-2	GT-1	GT-2	ST-1	ST-2	RST	FT
Echimidine	10.6±1.2	14.3±1.4	11.1±1.2	21.4±1.5	16.2±1.3	9.3±1.2	12.3±1.3	14.7±1.5	11.3±1.3	11.6±1.1	80.0±1.6	12.6±1.1	10.2±1.3	9.3±1.2
Erucifoline	<LOQ	<LOQ	<LOQ	<LOQ	22.6±1.3	22.5±1.1	<LOQ	23.0±1.1	<LOQ	<LOQ	22.5±1.5	<LOQ	22.5±1.7	23.4±1.5
Europine-N-oxide	<LOQ	<LOQ	<LOQ	<LOQ	<LOQ	<LOQ	<LOQ	<LOQ	<LOQ	<LOQ	6.7±0.7	<LOQ	<LOQ	<LOQ
Heliotrine	14.0±0.9	<LOQ	14.0±1.1	2.8±0.8	<LOQ	14.1±1.2	<LOQ	<LOQ	14.0±1.3	<LOQ	14.4±1.2	3.0±0.8	14.0±1.1	<LOQ
Heliotrine N-oxide	8.2±0.7	<LOQ	<LOQ	<LOQ	<LOQ	8.5±0.6	<LOQ	<LOQ	<LOQ	<LOQ	<LOQ	<LOQ	<LOQ	<LOQ
Jacobine	<LOQ	<LOQ	13.0±1.1	<LOQ	<LOQ	<LOQ	<LOQ	<LOQ	13.0±0.9	<LOQ	<LOQ	<LOQ	<LOQ	<LOQ
Lasiocarpine	1.5±0.4	0.6±0.2	3.6±0.4	0.5±0.1	0.6±0.1	<LOQ	0.4±0.1	0.5±0.1	4.6±0.5	0.6±0.1	<LOQ	0.5±0.1	1.7±0.2	0.6±0.1
Monocrotaline	<LOQ	<LOQ	<LOQ	9.6±0.9	<LOQ	242.8±1.1	<LOQ	<LOQ	<LOQ	<LOQ	29.7±0.9	<LOQ	<LOQ	<LOQ
Monocrotaline-N-oxide	2.4±0.6	2.7±0.3	4.1±0.8	3.7±0.5	<LOQ	3.1±0.7	2.0±0.3	<LOQ	2.6±0.4	2.9±0.5	<LOQ	<LOQ	<LOQ	2.3±0.4
Retrorsine	<LOQ	<LOQ	<LOQ	49.9±0.7	<LOQ	<LOQ	<LOQ	<LOQ	3.6±0.4	<LOQ	<LOQ	<LOQ	<LOQ	<LOQ
Retrorsine N-oxide	<LOQ	<LOQ	<LOQ	<LOQ	<LOQ	<LOQ	<LOQ	<LOQ	<LOQ	<LOQ	<LOQ	<LOQ	<LOQ	<LOQ
Senecionine	6.5±0.6	<LOQ	<LOQ	<LOQ	<LOQ	<LOQ	<LOQ	<LOQ	9.5±0.9	7.1±0.6	<LOQ	6.8±0.5	<LOQ	<LOQ
Senecionine N-oxide	<LOQ	<LOQ	<LOQ	<LOQ	<LOQ	<LOQ	<LOQ	<LOQ	8.4±0.5	8.6±0.6	<LOQ	7.9±0.7	<LOQ	<LOQ
Seneciphylline	10.7±0.9	10.8±1.1	<LOQ	<LOQ	<LOQ	<LOQ	<LOQ	<LOQ	12.8±1.1	<LOQ	<LOQ	<LOQ	10.8±0.8	<LOQ
Seneciphylline N-oxide	7.7±0.6	7.6±0.8	<LOQ	<LOQ	<LOQ	<LOQ	<LOQ	<LOQ	7.7±0.6	<LOQ	<LOQ	<LOQ	7.5±0.7	<LOQ
Senecivernine	<LOQ	<LOQ	<LOQ	<LOQ	<LOQ	<LOQ	<LOQ	<LOQ	<LOQ	<LOQ	<LOQ	<LOQ	<LOQ	<LOQ
Senecivernine N-oxide	<LOQ	<LOQ	<LOQ	<LOQ	<LOQ	<LOQ	<LOQ	<LOQ	<LOQ	<LOQ	<LOQ	<LOQ	<LOQ	<LOQ
Riddelline	<LOQ	16.0±1.1	49.5±1.3	41.9±1.1	1.8±0.5	<LOQ	1.6±0.4	<LOQ	<LOQ	<LOQ	9.3±0.8	<LOQ	2.0±0.5	<LOQ
Integerrimine	<LOQ	<LOQ	<LOQ	<LOQ	<LOQ	<LOQ	<LOQ	<LOQ	<LOQ	<LOQ	<LOQ	<LOQ	<LOQ	<LOQ
Atropine	<LOQ	<LOQ	<LOQ	<LOQ	<LOQ	<LOQ	<LOQ	29.3±1.3	<LOQ	482.3±1.6	<LOQ	<LOQ	<LOQ	<LOQ
Scopolamine	<LOQ	<LOQ	<LOQ	<LOQ	35.1±1.5	<LOQ	<LOQ	<LOQ	<LOQ	<LOQ	<LOQ	<LOQ	244.5±1.8	<LOQ
Intermedine+Lycopsamine+Indicine	86.6±1.8	144.5±1.7	<LOQ	<LOQ	<LOQ	296.0±1.7	91.2±1.6	112.2±1.5	<LOQ	268.6±1.4	96.4±1.5	<LOQ	<LOQ	<LOQ
Intermedine+Lycopsamine+Indicine-N-oxides	<LOQ	<LOQ	<LOQ	<LOQ	<LOQ	<LOQ	<LOQ	<LOQ	<LOQ	<LOQ	<LOQ	<LOQ	<LOQ	<LOQ

SM. 5. Data of samples for PAs and TAs

Compound	CT-1	CT-2	CT-3	CT-4	FC	PM-1	PM-2	PM-3	PM-4	PM-5	PM-6	PM-7	FPM	LT-1	LT-2
Echimidine	9.3±0.9	8.8±1.0	2.0±0.4	1.8±0.4	9.5±1.1	19.3±1.4	13.6±1.1	18.1±0.8	1.7±0.5	<LOQ	<LOQ	1.8±0.4	9.3±0.9	9.6±0.8	16.7±1.1
Erucifoline	22.3±1.2	22.9±1.1	4.8±0.8	4.8±0.8	41.0±1.4	126.2±1.4	70.3±1.1	67.2±1.5	5.4±0.8	4.5±0.7	4.7±0.8	4.5±0.80.9	59.2±	<LOQ	23.0±0.9
Europine-N-oxide	<LOQ	<LOQ	<LOQ	<LOQ	<LOQ	<LOQ	<LOQ	<LOQ	<LOQ	<LOQ	<LOQ	<LOQ	<LOQ	<LOQ	<LOQ
Heliotrine	<LOQ	14.5±0.8	<LOQ	<LOQ	2.9±0.4	<LOQ	<LOQ	<LOQ	<LOQ	<LOQ	<LOQ	<LOQ	14.8±0.8	4.2±0.6	14.5±1.5
Heliotrine N-oxide	<LOQ	8.6±0.7	2.7±0.4	<LOQ	<LOQ	<LOQ	<LOQ	<LOQ	2.5±0.4	2.9±0.5	1.7±0.5	1.8±0.3	12.9±0.6	1.7±0.4	8.6±0.8
Jacobine	<LOQ	13.2±0.9	<LOQ	12.5±	<LOQ	<LOQ	<LOQ	<LOQ	<LOQ	<LOQ	12.8±	12.6±	<LOQ	2.7±0.5	<LOQ
Lasiocarpine	0.5±	2.0±0.4	4.3±	2.5±0.5	1.7±0.3	112.8±1.5	59.7±0.9	96.4±1.1	2.1±0.3	6.7±0.8	2.1±0.4	2.1±0.4	9.7±0.9	0.8±0.4	1.6±0.7
Monocrotaline	<LOQ	136.7±1.5	<LOQ	<LOQ	35.8±1.4	<LOQ	<LOQ	<LOQ	<LOQ	<LOQ	<LOQ	<LOQ	<LOQ	<LOQ	<LOQ
Monocrotaline-N-oxide	<LOQ	4.2±0.8	2.0±0.4	<LOQ	410.1±1.8	<LOQ	<LOQ	<LOQ	3.9±0.6	2.2±0.4	5.8±0.7	6.0±0.6	<LOQ	2.2±0.6	2.8±0.6
Retrorsine	<LOQ	3.4±0.6	<LOQ	3.1±0.5	<LOQ	<LOQ	<LOQ	16.3±0.9	<LOQ	<LOQ	3.2±0.5	3.2±0.7	<LOQ	<LOQ	<LOQ
Retrorsine N-oxide	<LOQ	<LOQ	<LOQ	<LOQ	265.1±1.6	<LOQ	<LOQ	<LOQ	56.9±1.3	4.0±0.8	<LOQ	<LOQ	<LOQ	<LOQ	<LOQ
Senecionine	<LOQ	7.7±0.7	8.0±0.6	6.7±0.6	<LOQ	19.5±0.9	18.2±1.3	23.3±1.1	7.4±0.8	7.3±0.6	7.7±0.4	6.9±0.6	21.6±0.8	<LOQ	<LOQ
Senecionine N-oxide	<LOQ	8.3±0.9	<LOQ	<LOQ	<LOQ	85.1±1.5	60.7±1.8	80.2±0.9	83.4±1.2	38.4±1.6	68.3±1.5	132.2±1.1	163.7±1.6	<LOQ	<LOQ
Seneciphylline	<LOQ	10.8±1.1	<LOQ	<LOQ	30.7±0.9	<LOQ	<LOQ	<LOQ	<LOQ	<LOQ	<LOQ	<LOQ	<LOQ	<LOQ	10.8±0.7
Seneciphylline N-oxide	<LOQ	8.2±0.5	7.8±0.7	7.9±0.4	22.8±0.9	105.8±1.1	52.7±1.3	49.8±1.4	8.3±0.8	7.6±0.6	8.6±0.7	7.9±0.6	42.3±0.8	<LOQ	8.0±0.6
Senecivernine	<LOQ	<LOQ	<LOQ	<LOQ	5.4±0.7	<LOQ	5.0±0.8	3.6±0.5	<LOQ	<LOQ	<LOQ	<LOQ	<LOQ	<LOQ	<LOQ
Senecivernine N-oxide	<LOQ	<LOQ	<LOQ	<LOQ	<LOQ	63.6±1.2	38.9±0.9	58.0±1.5	<LOQ	<LOQ	<LOQ	<LOQ	38.1±1.1	<LOQ	<LOQ
Riddelline	24.6±0.8	<LOQ	<LOQ	<LOQ	189.1±1.7	15.7±1.8	<LOQ	<LOQ	<LOQ	<LOQ	<LOQ	<LOQ	<LOQ	<LOQ	21.4±0.9
Integerrimine	<LOQ	<LOQ	<LOQ	<LOQ	<LOQ	<LOQ	<LOQ	<LOQ	<LOQ	<LOQ	<LOQ	<LOQ	<LOQ	<LOQ	<LOQ
Atropine	559.2±1.8	<LOQ	<LOQ	<LOQ	<LOQ	<LOQ	<LOQ	<LOQ	<LOQ	<LOQ	<LOQ	<LOQ	<LOQ	<LOQ	<LOQ
Scopolamine	<LOQ	<LOQ	<LOQ	<LOQ	<LOQ	<LOQ	<LOQ	<LOQ	<LOQ	<LOQ	<LOQ	<LOQ	<LOQ	<LOQ	<LOQ
Intermedine+Lycopsamine+Indicine	79.0±1.3	36.3±1.1	<LOQ	<LOQ	40.4±1.3	<LOQ	<LOQ	66.3±1.4	101.2±1.5	<LOQ	<LOQ	<LOQ	109.2±1.4	<LOQ	166.3±1.5
Intermedine+Lycopsamine+Indicine-N-oxides	<LOQ	<LOQ	<LOQ	<LOQ	<LOQ	<LOQ	<LOQ	<LOQ	<LOQ	<LOQ	<LOQ	<LOQ	<LOQ	<LOQ	27.1±1.2

SM. 5. Data of samples for PAs and TAs

Compound	T-1	T-2	T-3	T-4	T-5	T-6	T-7	T-8	T-9	T-10	T-11	T-12	T-13	T-14	T-15
Echimidine	1.8±0.6	1.8±0.4	1.9±0.4	<LOQ	1.7±0.3	1.8±0.4	<LOQ	<LOQ	<LOQ	<LOQ	11.9±1.4	<LOQ	<LOQ	<LOQ	<LOQ
Erucifoline	4.6±1.1	4.6±0.8	4.7±0.8	4.9±0.8	5.1±0.6	4.8±0.6	4.6±0.5	4.7±0.7	<LOQ	<LOQ	<LOQ	<LOQ	<LOQ	<LOQ	<LOQ
Europine-N-oxide	<LOQ	<LOQ	<LOQ	<LOQ	<LOQ	<LOQ	<LOQ	<LOQ	<LOQ	<LOQ	<LOQ	<LOQ	<LOQ	<LOQ	<LOQ
Heliotrine	<LOQ	<LOQ	<LOQ	<LOQ	<LOQ	<LOQ	<LOQ	<LOQ	<LOQ	<LOQ	<LOQ	<LOQ	<LOQ	<LOQ	<LOQ
Heliotrine N-oxide	<LOQ	1.7±0.4	1.7±0.6	1.6±0.4	1.7±0.5	<LOQ	<LOQ	<LOQ	<LOQ	<LOQ	<LOQ	<LOQ	<LOQ	<LOQ	<LOQ
Jacobine	92.2±1.6	<LOQ	<LOQ	57.3±1.1	13.1±1.1	<LOQ	<LOQ	<LOQ	<LOQ	<LOQ	<LOQ	<LOQ	<LOQ	<LOQ	<LOQ
Lasiocarpine	2.4±0.7	1.9±0.3	2.2±0.6	3.3±0.8	3.3±0.7	256.7±1.4	<LOQ	<LOQ	3.7±0.6	10.9±1.3	5.4±0.8	3.3±0.7	11.0±1.1	2.8±0.8	13.2±1.4
Monocrotaline	<LOQ	<LOQ	<LOQ	<LOQ	<LOQ	<LOQ	<LOQ	<LOQ	<LOQ	<LOQ	<LOQ	<LOQ	<LOQ	<LOQ	<LOQ
Monocrotaline-N-oxide	2.0±0.4	3.1±0.6	5.5±0.8	<LOQ	2.4±0.7	2.3±0.6	<LOQ	140.2±1.1	7.1±0.7	5.2±	5.2±0.8	5.6±0.8	6.2±0.7	7.8±1.0	22.3±0.9
Retrorsine	3.4±0.8	3.5±0.7	3.2±0.6	3.3±0.5	3.6±0.7	16.5±1.1	<LOQ	<LOQ	<LOQ	22.7±	<LOQ	<LOQ	15.6±1.1	<LOQ	15.4±1.2
Retrorsine N-oxide	<LOQ	<LOQ	<LOQ	<LOQ	<LOQ	<LOQ	<LOQ	<LOQ	<LOQ	<LOQ	<LOQ	<LOQ	<LOQ	<LOQ	<LOQ
Senecionine	8.2±1.1	6.9±1.2	7.6±0.8	7.2±0.7	7.3±0.8	7.5±0.6	<LOQ	<LOQ	<LOQ	<LOQ	<LOQ	<LOQ	<LOQ	<LOQ	<LOQ
Senecionine N-oxide	8.6±0.8	<LOQ	<LOQ	8.5±0.9	8.7±0.8	8.9±0.7	<LOQ	<LOQ	<LOQ	<LOQ	<LOQ	<LOQ	<LOQ	<LOQ	<LOQ
Seneciphylline	10.9±0.9	10.7±1.3	10.7±0.9	<LOQ	10.0±1.1	<LOQ	<LOQ	<LOQ	12.1±1.3	11.4±0.9	14.0±0.8	<LOQ	12.5±1.1	<LOQ	<LOQ
Seneciphylline N-oxide	7.6±0.6	7.8±0.7	7.5±0.5	<LOQ	7.6±0.6	<LOQ	<LOQ	<LOQ	9.6±1.1	9.0±1.1	9.4±0.8	7.3±0.7	10.1±0.9	9.0±1.2	7.4±1.1
Senecivernine	<LOQ	<LOQ	<LOQ	<LOQ	<LOQ	<LOQ	<LOQ	<LOQ	<LOQ	<LOQ	<LOQ	<LOQ	<LOQ	<LOQ	<LOQ
Senecivernine N-oxide	<LOQ	<LOQ	<LOQ	<LOQ	<LOQ	<LOQ	<LOQ	<LOQ	1.9±0.8	20.1±1.1	<LOQ	<LOQ	13.0±1.2	<LOQ	12.5±1.3
Riddelline	<LOQ	<LOQ	<LOQ	<LOQ	<LOQ	<LOQ	<LOQ	<LOQ	<LOQ	5.1±0.7	<LOQ	<LOQ	4.9±0.8	<LOQ	4.1±0,8
Integerrimine	<LOQ	<LOQ	<LOQ	<LOQ	<LOQ	<LOQ	<LOQ	<LOQ	<LOQ	1.6±0.5	<LOQ	1.7±0.6	<LOQ	<LOQ	<LOQ
Atropine	<LOQ	<LOQ	<LOQ	<LOQ	<LOQ	<LOQ	<LOQ	<LOQ	<LOQ	<LOQ	<LOQ	<LOQ	<LOQ	<LOQ	<LOQ
Scopolamine	<LOQ	<LOQ	<LOQ	<LOQ	<LOQ	<LOQ	<LOQ	<LOQ	<LOQ	<LOQ	<LOQ	<LOQ	<LOQ	<LOQ	<LOQ
Intermedine+Lycopsamine+Indicine	<LOQ	<LOQ	<LOQ	<LOQ	<LOQ	137.5±1.1	<LOQ	<LOQ	<LOQ	<LOQ	<LOQ	<LOQ	<LOQ	<LOQ	<LOQ
Intermedine+Lycopsamine+Indicine-N-oxides	<LOQ	<LOQ	<LOQ	<LOQ	<LOQ	<LOQ	<LOQ	<LOQ	<LOQ	<LOQ	<LOQ	<LOQ	<LOQ	<LOQ	<LOQ

SM-6. Regression equations and R^2 values of solvent and matrix-matched calibration for thyme matrix.

Compound Name	Regression equation solvent	R^2 solvent	Regression equation Thyme matrix	R^2 Thyme matrix	ME %
Echimidine	y = 113975x - 188691	0.9996	y = 130018x - 105872	0.9990	14.1
Erucifoline	y = 98243x - 438608	0.9997	y = 94336x + 125070	0.9991	-4.0
Europine	y = 75299x + 40588	0.9998	y = 81865x + 118989	0.9993	8.7
Europine *N-oxide*	y = 75711x + 39711	0.9999	y = 72749x + 63501	0.9993	-3.9
Heliotrine	y = 92882x - 259552	0.9994	y = 90800x + 231539	0.9992	-2.2
Heliotrine *N-oxide*	y = 116893x - 192370	0.9995	y = 105800x - 19053	0.9991	-9.5
Jacobine	y = 83888x - 206710	0.9994	y = 73993x + 40982	0.9998	-11.8
Jacobine *N-oxide*	y = 49715x + 220560	0.9992	y = 51085x - 13067	0.9996	2.8
Lasiocarpine	y = 228585x - 70155	0.9997	y = 224226x + 465188	0.9998	-1.9
Lasiocarpine *N-oxide*	y = 180097x + 785864	0.9992	y = 199127x + 286134	0.9993	10.6
Monocrotaline	y = 84062x + 247397	0.9998	y = 87480x + 92711	0.9995	4.1
Monocrotaline *N-oxide*	y = 49678x - 16030	0.9999	y = 48842x + 229527	0.9993	-1.7
Retrorsine	y = 66827x - 38398	0.9996	y = 65695x + 25950	0.9994	-1.7
Retrorsine *N-oxide*	y = 43255x + 203752	0.9997	y = 47489x + 201319	0.9994	9.8
Senecionine	y = 86527x - 112901	0.9998	y = 90810x + 49113	0.9992	4.9
Senecionine *N-oxide*	y = 140271x - 217910	0.9997	y = 145281x + 152159	0.9995	3.6
Seneciphylline	y = 130884x - 275878	0.9995	y = 111449x + 82848	0.9991	-14.8
Seneciphylline *N-oxide*	y = 103691x - 152094	0.9997	y = 108493x + 265026	0.9995	4.6
Senecivernine	y = 157349x + 277937	0.9998	y = 136975x - 313033	0.9996	-12.9
Senecivernine *N-oxide*	y = 142711x + 375742	0.9997	y = 144947x + 102977	0.9991	1.6
Riddelline	y = 75303x + 122694	0.9998	y = 73530x + 36639	0.9994	-2.4
Riddelline *N-oxide*	y = 56263x + 372724	0.9995	y = 53243x + 280834	0.9992	-5.4
Senkirkine	y = 200533x + 439894	0.9998	y = 209931x + 176267	0.9993	4.7
Trichodesmine	y = 121840x + 173831	0.9997	y = 123528x - 39096	0.9994	1.4
Integerrimine	y = 100282x + 203926	0.9996	y = 111904x - 42477	0.9999	11.6
Atropine	y = 136867x + 284758	0.9994	y = 130627x + 509070	0.9993	-4.6
Scopolamine	y = 66561x - 284524	0.9990	y = 68241x + 172645	0.9997	2.5
Intermedine<br>Lycopsamine<br>indicine	y = 200383x - 216672	0.9991	y=179751x+117658	0.9993	**-10,3**
Intermedine-N-oxides<br>Lycopsamine-N-oxides<br>indicine-N-oxides	y = 141661x + 113442	0.9998	y=124686x-98765	0.9994	**-12,0**

SM-7. Regression equations and R^2 values of solvent and matrix-matched calibration for tea matrix.

Compound Name	Regression equation solvent	R2 solvent	Regression equation Tea matrix	R2 tea matrix	ME %
Echimidine	y = 113975x - 188691	0.9996	y = 116551x - 369541	0.9994	2.3
Erucifoline	y = 98243x - 438608	0.9997	y = 91670x + 157384	0.9992	-6.7
Europine	y = 75299x + 40588	0.9998	y = 84260x + 15405	0.9998	11.9
Europine N-oxide	y = 75711x + 39711	0.9999	y = 78119x - 127569	0.9993	3.2
Heliotrine	y = 92882x - 259552	0.9994	y = 96457x - 252269	0.9995	3.8
Heliotrine N-oxide	y = 116893x - 192370	0.9995	y = 105151x + 63450	0.9989	-10.0
Jacobine	y = 83888x - 206710	0.9994	y = 82989x - 427486	0.9992	-1.2
Jacobine N-oxide	y = 49715x + 220560	0.9992	y = 44472x + 139181	0.9996	-10.5
Lasiocarpine	y = 228585x - 70155	0.9997	y = 209309x - 170941	0.9994	-8.4
Lasiocarpine N-oxide	y = 180097x + 785864	0.9992	y = 174670x + 534396	0.9994	-3.0
Monocrotaline	y = 84062x + 247397	0.9998	y = 70466x - 32801	0.9992	-16.2
Monocrotaline N-oxide	y = 49678x - 16030	0.9999	y = 43309x + 232532	0.9991	-12.8
Retrorsine	y = 66827x - 38398	0.9996	y = 58804x + 15618	0.9996	-12.0
Retrorsine N-oxide	y = 43255x + 203752	0.9997	y = 36518x + 183015	0.9997	-15.6
Senecionine	y = 86527x - 112901	0.9998	y = 95693x + 409119	0.9998	10.6
Senecionine N-oxide	y = 140271x - 217910	0.9997	y = 123514x + 135342	0.9991	-11.9
Seneciphylline	y = 130884x - 275878	0.9995	y = 109798x - 203362	0.9997	-16.1
Seneciphylline N-oxide	y = 103691x - 152094	0.9997	y = 95836x - 95804	0.9995	-7.6
Senecivernine	y = 157349x + 277937	0.9998	y = 130974x - 462829	0.9995	-16.8
Senecivernine N-oxide	y = 142711x + 375742	0.9997	y = 123767x - 9168,5	0.9996	-13.3
Riddelline	y = 75303x + 122694	0.9998	y = 65911x - 24078	0.9995	-12.5
Riddelline N-oxide	y = 56263x + 372724	0.9995	y = 47912x + 233929	0.9993	-14.8
Senkirkine	y = 200533x + 439894	0.9998	y = 183395x + 131230	0.9992	-8.5
Trichodesmine	y = 121840x + 173831	0.9997	y = 107131x - 171418	0.9994	-12.1
Integerrimine	y = 100282x + 203926	0.9996	y = 105128x - 276675	0.9992	4.8
Atropine	y = 136867x + 284758	0.9994	y = 121633x + 535166	0.9995	-11.12
Scopolamine	y = 66561x - 284524	0.9990	y = 63755x + 148479	0.9995	-4.2
Intermedine<br>Lycopsamine<br>Indicine	y = 200383x - 216672	0.9991	y=219853x-182331	0.9992	9.7
Intermedine-N-oxides<br>Lycopsamine-N-oxides<br>Indicine-N-oxides	y = 141661x + 113442	0.9998	y=128345x-634531	0.9995	-9.4

SM-8. Regression equations and R^2 values of solvent and matrix-matched calibration for chamomile matrix.

Compound Name	Regression equation solvent	R^2 solvent	Regression equation Chamomile matrix	R^2 Chamomile matrix	ME %
Echimidine	y = 113975x - 188691	0.9996	y = 120206x + 106757	0.9995	5.5
Erucifoline	y = 98243x - 438608	0.9997	y = 97696x + 318981	0.9993	-0.6
Europine	y = 75299x + 40588	0.9998	y = 76582x - 49794	0.9993	1.7
Europine *N-oxide*	y = 75711x + 39711	0.9999	y = 82260x - 57482	0.9995	8.6
Heliotrine	y = 92882x - 259552	0.9994	y = 94099x + 92346	0.9995	1.3
Heliotrine *N-oxide*	y = 116893x - 192370	0.9995	y = 112571x + 88206	0.9993	-3.7
Jacobine	y = 83888x - 206710	0.9994	y = 82112x - 129429	0.9994	-2.1
Jacobine *N-oxide*	y = 49715x + 220560	0.9992	y = 48746x + 22664	0.9993	-1.9
Lasiocarpine	y = 228585x - 70155	0.9997	y = 223975x + 514455	0.9995	-2.0
Lasiocarpine *N-oxide*	y = 180097x + 785864	0.9992	y = 192799x + 909758	0.9991	7.1
Monocrotaline	y = 84062x + 247397	0.9998	y = 83329x - 46080	0.9997	-0.9
Monocrotaline *N-oxide*	y = 49678x - 16030	0.9999	y = 48500x + 160755	0.9993	-2.4
Retrorsine	y = 66827x - 38398	0.9996	y = 66205x + 124379	0.9994	-0.9
Retrorsine *N-oxide*	y = 43255x + 203752	0.9997	y = 45105x + 132353	0.9997	4.3
Senecionine	y = 86527x - 112901	0.9998	y = 88217x + 306978	0.9997	2.0
Senecionine *N-oxide*	y = 140271x - 217910	0.9997	y = 142261x + 193553	0.9991	1.4
Seneciphylline	y = 130884x - 275878	0.9995	y = 132115x + 44839	0.9994	0.9
Seneciphylline *N-oxide*	y = 103691x - 152094	0.9997	y = 100206x + 124678	0.9996	-3.4
Senecivernine	y = 157349x + 277937	0.9998	y = 137254x - 60699	0.9995	-12.8
Senecivernine *N-oxide*	y = 142711x + 375742	0.9997	y = 131801x + 491175	0.9993	-7.6
Riddelline	y = 75303x + 122694	0.9998	y = 76095x + 2299	0.9996	1.1
Riddelline *N-oxide*	y = 56263x + 372724	0.9995	y = 57878x + 147265	0.9993	2.9
Senkirkine	y = 200533x + 439894	0.9998	y = 203213x + 531930	0.9991	1.3
Trichodesmine	y = 121840x + 173831	0.9997	y = 114446x + 49918	0.9994	-6.1
Integerrimine	y = 100282x + 203926	0.9996	y = 113379x + 80168	0.9995	13.1
Atropine	y = 136867x + 284758	0.9994	y = 145663x + 444731	0.9992	6.4
Scopolamine	y = 66561x - 284524	0.9990	y = 69474x + 314841	0.9994	4.4
Intermedine<br>Lycopsamine<br>Indicine	y = 200383x - 216672	0.9991	y=186456x-187654	0.9992	-7.0
Intermedine-N-oxides<br>Lycopsamine-N-oxides<br>Indicine-N-oxides	y = 141661x + 113442	0.9998	y=133783x-100653	0.9997	-5.6

SM-9. Regression equations and R^2 values of solvent and matrix-matched calibration for peppermint matrix.

Compound Name	Regression equation solvent	R^2 solvent	Regression equation Peppermint matrix	R^2 Peppermint matrix	ME %
Echimidine	y = 113975x - 188691	0.9996	y = 107622x - 7404	0.9993	-5.6
Erucifoline	y = 98243x - 438608	0.9997	y = 97130x - 27755	0.9992	-1.1
Europine	y = 75299x + 40588	0.9998	y = 73798x + 125245	0.9997	-2.0
Europine *N-oxide*	y = 75711x + 39711	0.9999	y = 74353x - 132791	0.9994	-1.8
Heliotrine	y = 92882x - 259552	0.9994	y = 91871x + 54324	0.9991	-1.1
Heliotrine *N-oxide*	y = 116893x - 192370	0.9995	y = 108502x + 152924	0.9993	-7.2
Jacobine	y = 83888x - 206710	0.9994	y = 81323x + 28209	0.9996	-3.1
Jacobine *N-oxide*	y = 49715x + 220560	0.9992	y = 50736x - 64854	0.9994	2.1
Lasiocarpine	y = 228585x - 70155	0.9997	y = 215206x + 470211	0.9995	-5.9
Lasiocarpine *N-oxide*	y = 180097x + 785864	0.9992	y = 174886x + 438032	0.9993	-2.9
Monocrotaline	y = 84062x + 247397	0.9998	y = 83411x - 40794	0.9997	-0.8
Monocrotaline *N-oxide*	y = 49678x - 16030	0.9999	y = 50781x + 128030	0.9992	2.2
Retrorsine	y = 66827x - 38398	0.9996	y = 67174x + 70765	0.9994	0.5
Retrorsine *N-oxide*	y = 43255x + 203752	0.9997	y = 44491x + 166652	0.9993	2.9
Senecionine	y = 86527x - 112901	0.9998	y = 85194x + 22375	0.9992	-1.5
Senecionine *N-oxide*	y = 140271x - 217910	0.9997	y = 134139x + 622172	0.9993	-4.4
Seneciphylline	y = 130884x - 275878	0.9995	y = 128518x + 252379	0.9996	-1.8
Seneciphylline *N-oxide*	y = 103691x - 152094	0.9997	y = 99968x + 358972	0.9995	-3.6
Senecivernine	y = 157349x + 277937	0.9998	y = 144281x - 37270	0.9994	-8.3
Senecivernine *N-oxide*	y = 142711x + 375742	0.9997	y = 136106x + 230928	0.9993	-4.6
Riddelline	y = 75303x + 122694	0.9998	y = 74076x + 120862	0.9997	-1.6
Riddelline *N-oxide*	y = 56263x + 372724	0.9995	y = 57993x + 72050	0.9992	3.1
Senkirkine	y = 200533x + 439894	0.9998	y = 196751x - 65520	0.9998	-1.9
Trichodesmine	y = 121840x + 173831	0.9997	y = 119038x - 314307	0.9991	-2.3
Integerrimine	y = 100282x + 203926	0.9996	y = 101393x - 218223	0.9995	1.1
Atropine	y = 136867x + 284758	0.9994	y = 151098x + 585576	0.9995	10.4
Scopolamine	y = 66561x - 284524	0.9990	y = 67106x + 268367	0.9993	0.8
Intermedine<br>Lycopsamine<br>Indicine	y = 200383x - 216672	0.9991	y=207458x-17658	0.9994	3.5
Intermedine-N-oxides<br>Lycopsamine-N-oxides<br>Indicine-N-oxides	y = 141661x + 113442	0.9998	y=132587x-78654	0.9996	-6.4

SM-10. Score and loading plots of thyme samples.

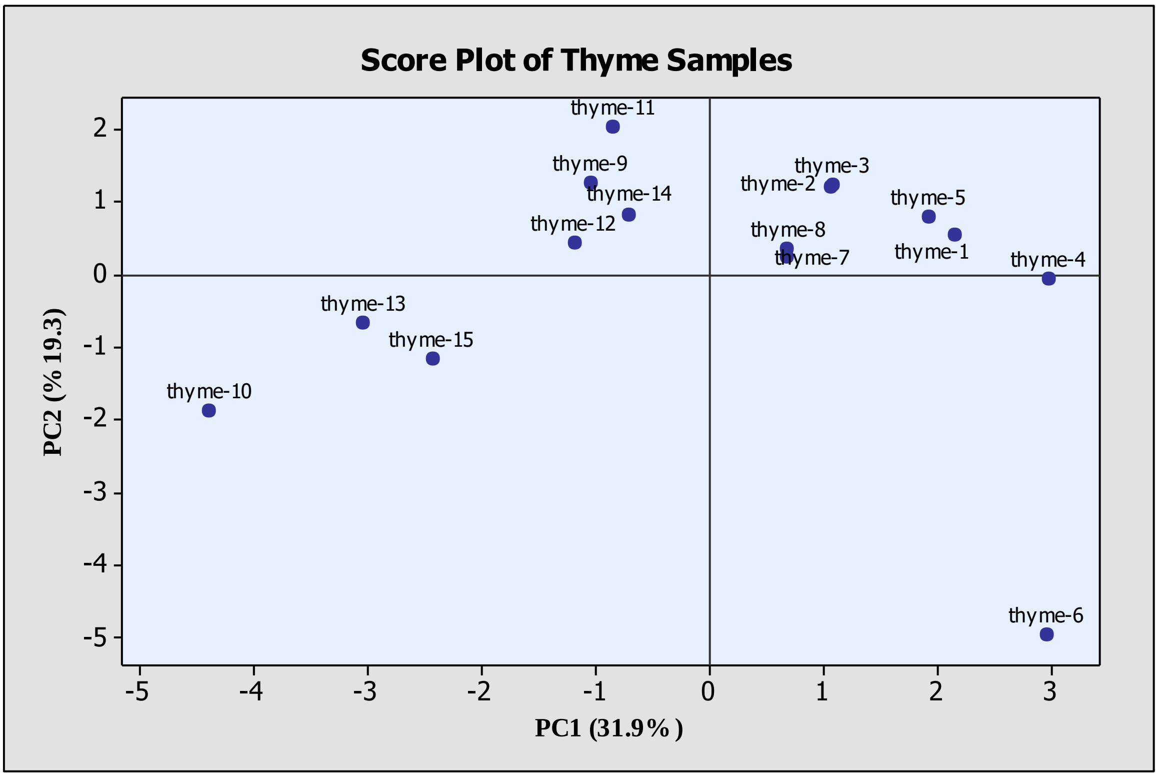

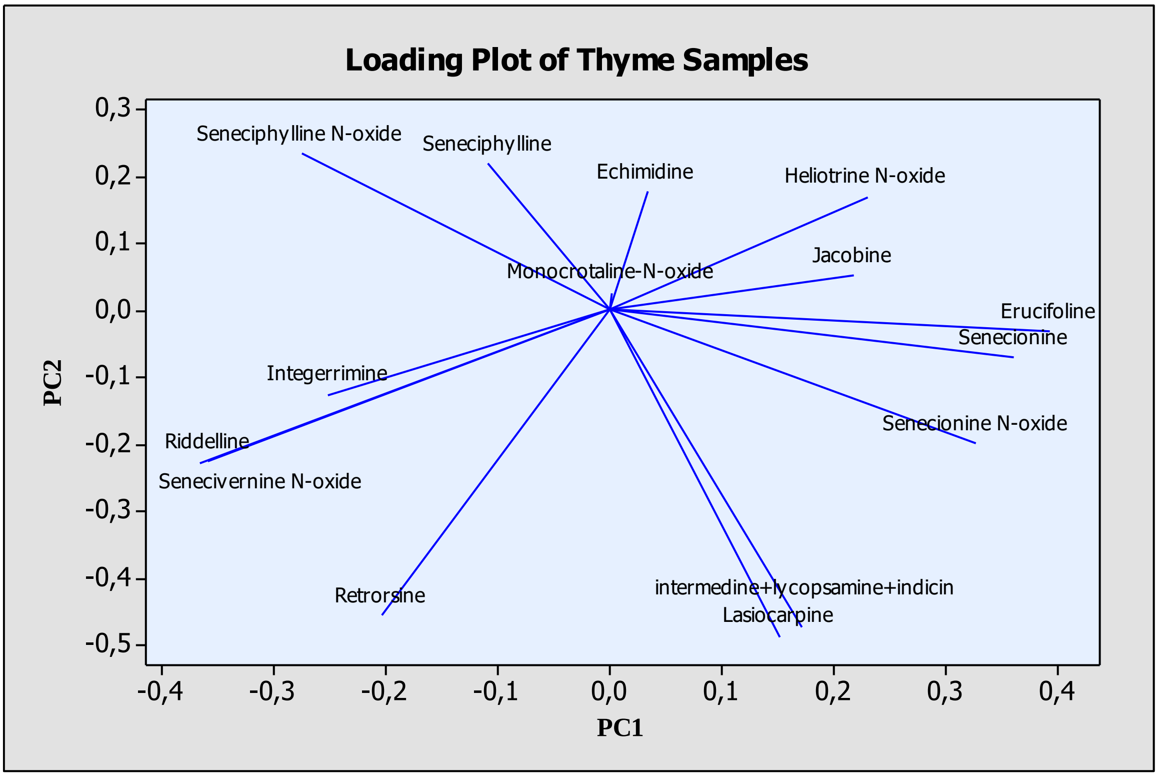

SM-11. Score and loading plots of tea samples.

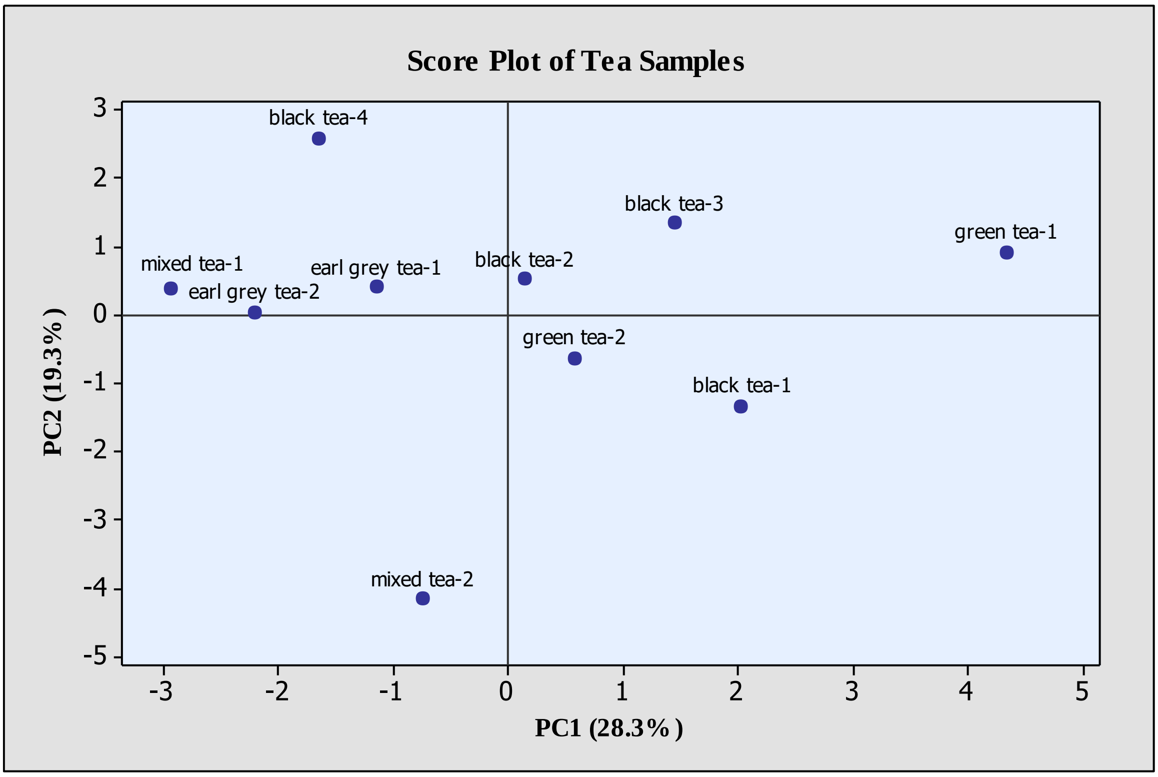

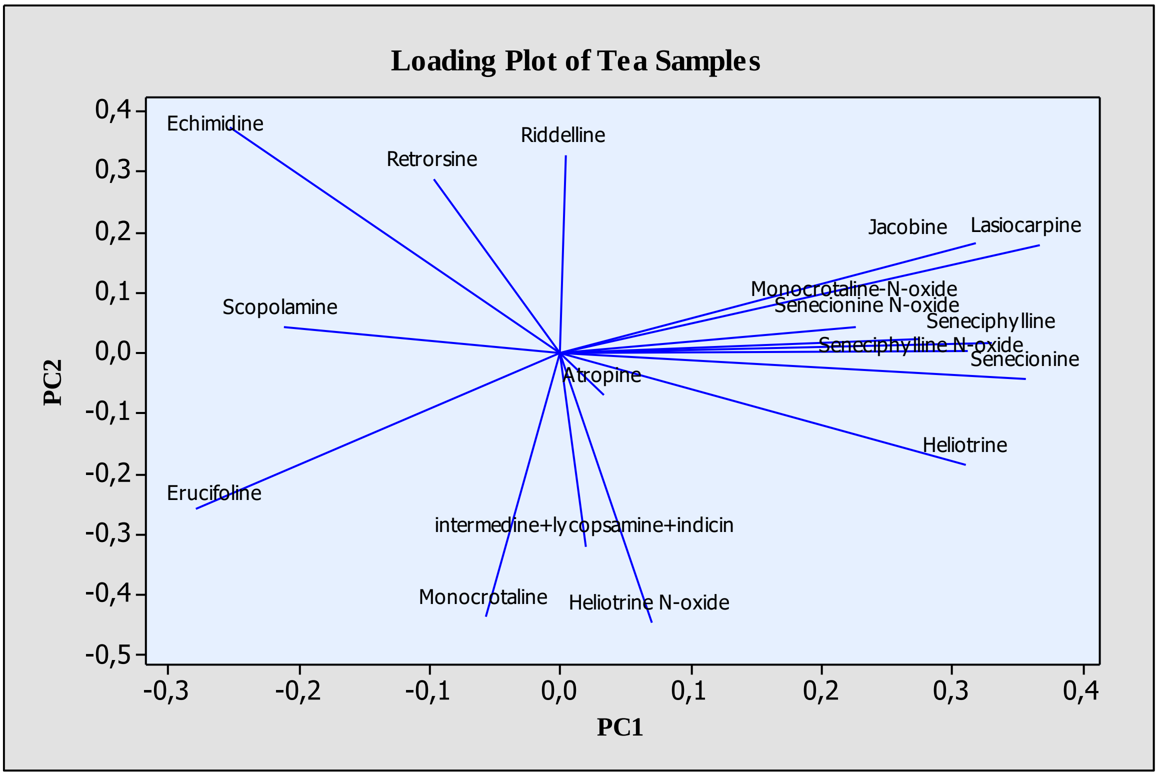

SM-12. Score and loading plots of chamomile samples.

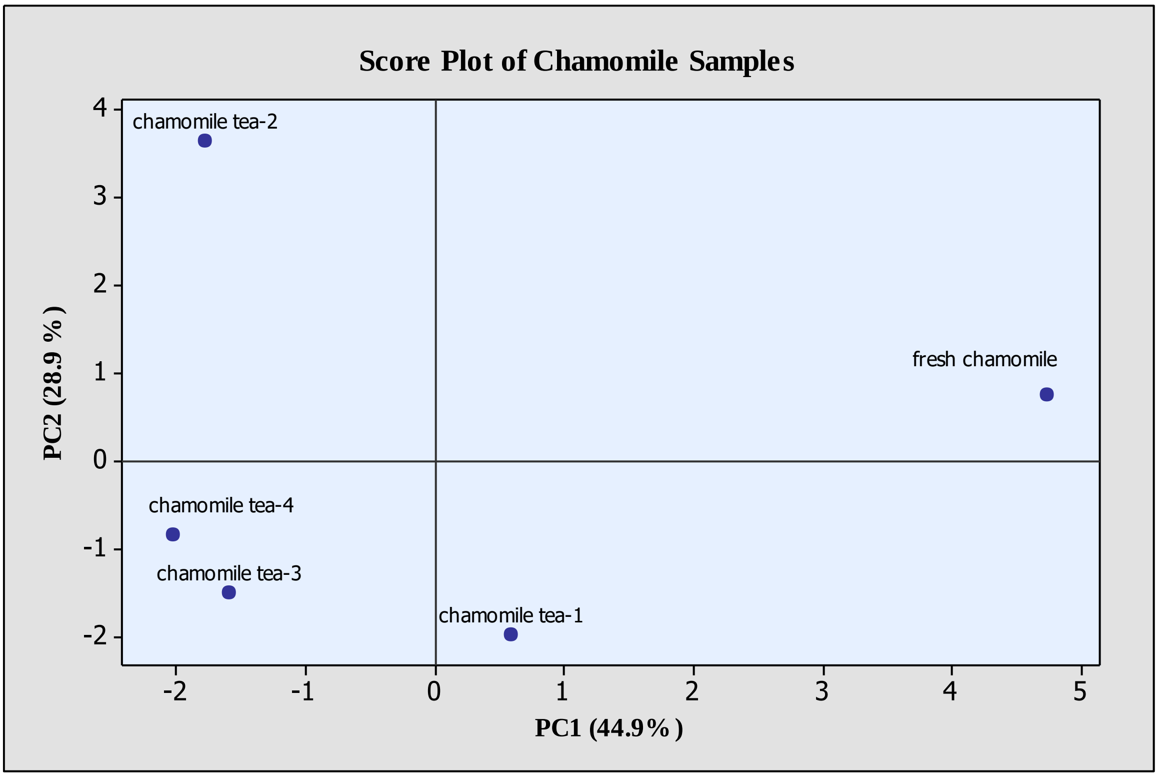

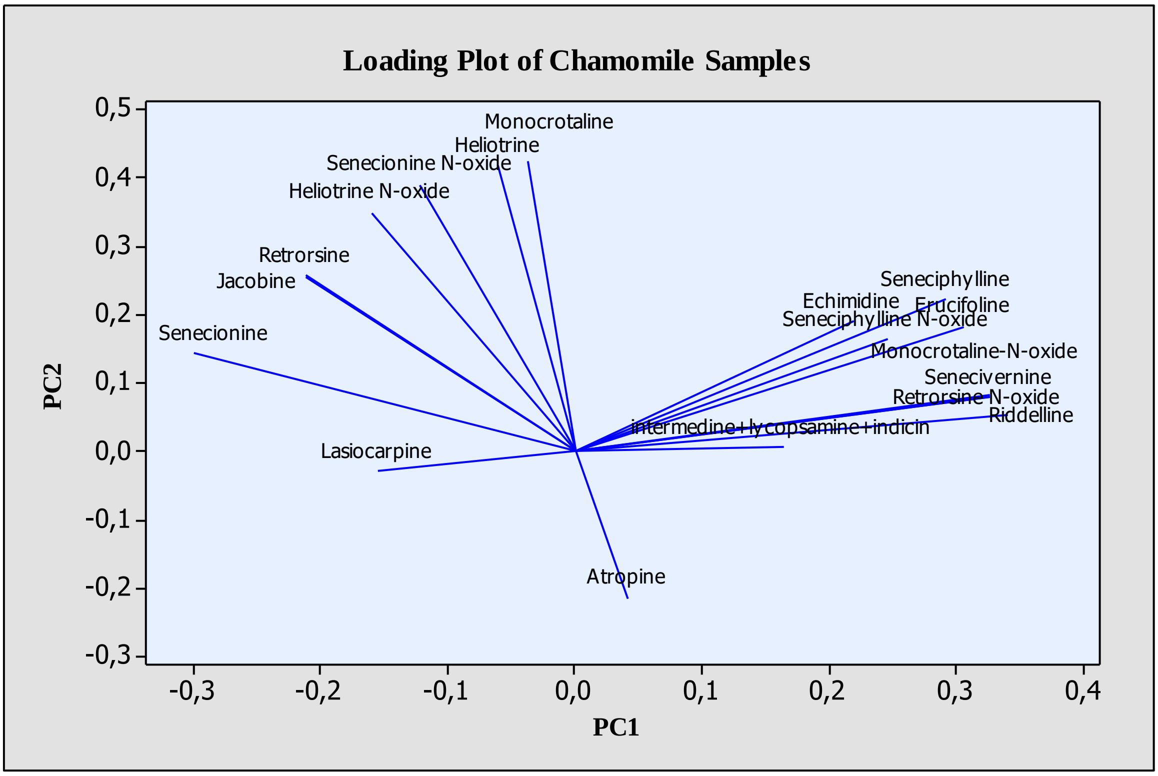

SM-13. Score and loading plots of peppermint samples.

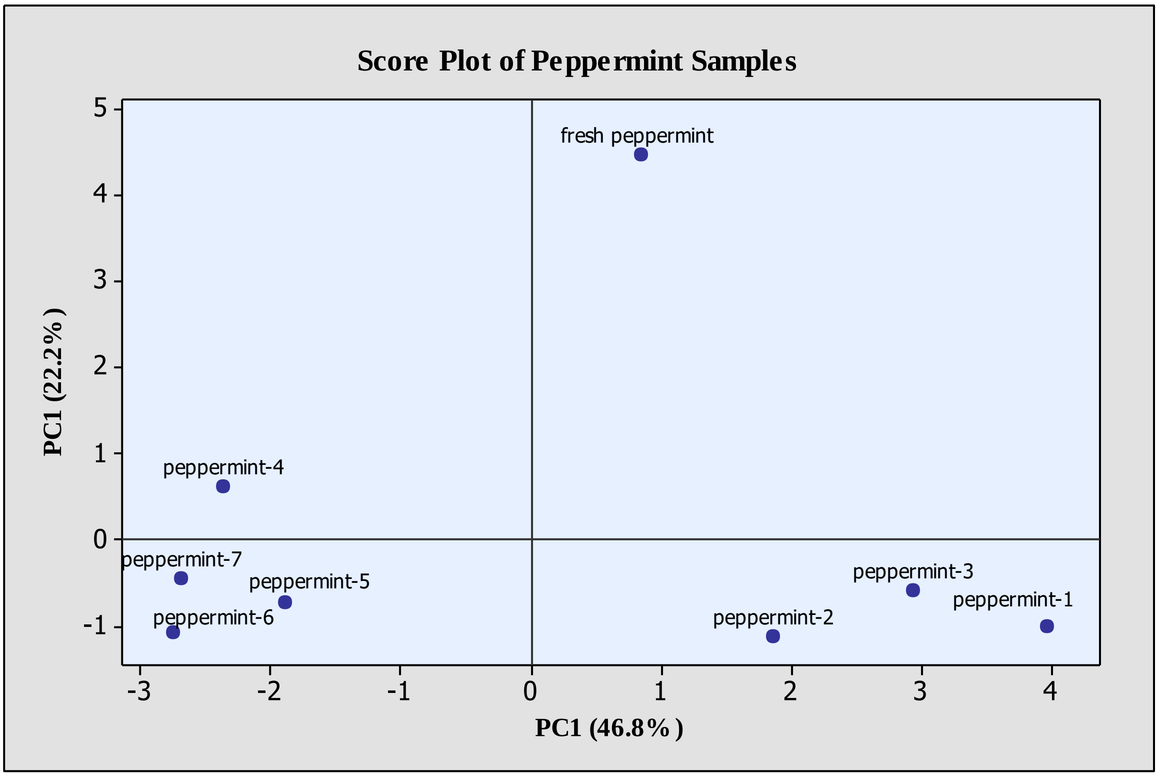

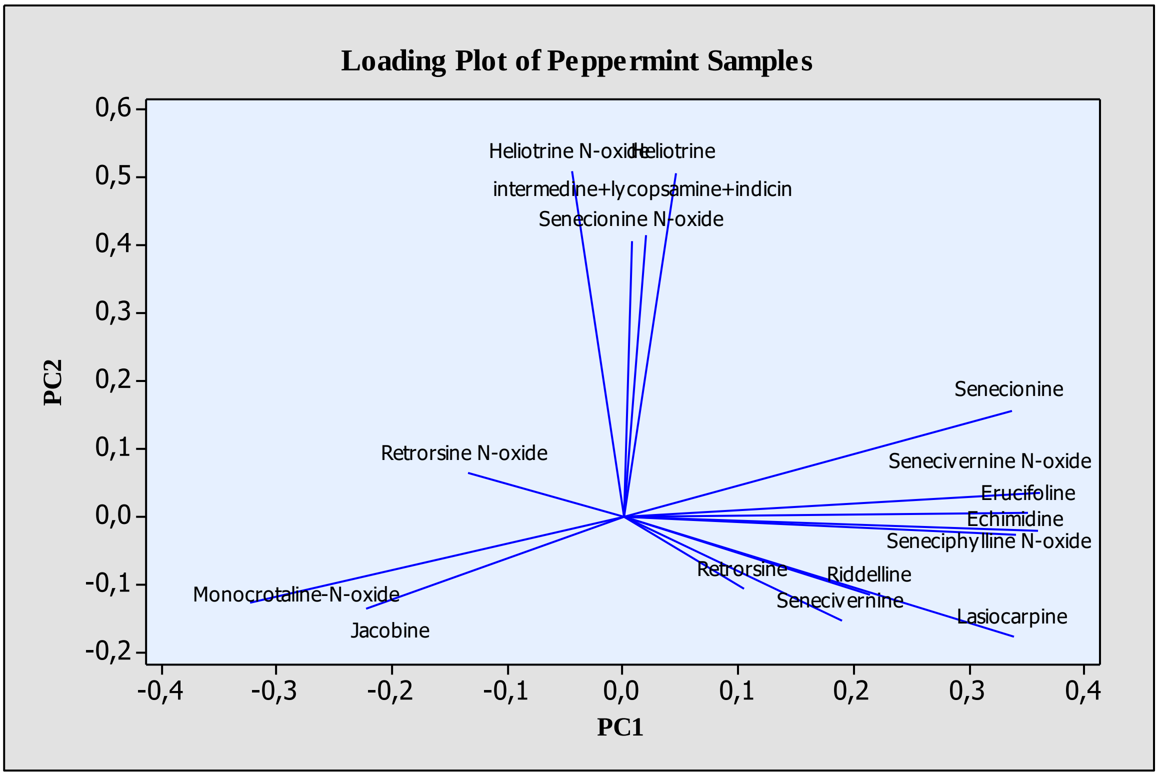

Supplement: Supplementary file 1 [file foods-12-03572-s001.zip › foods-2599029-supplementary.pdf]
